# Projections of Water Stress Based on an Ensemble of Socioeconomic Growth and Climate Change Scenarios: A Case Study in Asia

**DOI:** 10.1371/journal.pone.0150633

**Published:** 2016-03-30

**Authors:** Charles Fant, C. Adam Schlosser, Xiang Gao, Kenneth Strzepek, John Reilly

**Affiliations:** Joint Program on the Science and Policy of Global Change, Massachusetts Institute of Technology, Cambridge, MA, United States of America; University of Washington, UNITED STATES

## Abstract

The sustainability of future water resources is of paramount importance and is affected by many factors, including population, wealth and climate. Inherent in current methods to estimate these factors in the future is the uncertainty of their prediction. In this study, we integrate a large ensemble of scenarios—internally consistent across economics, emissions, climate, and population—to develop a risk portfolio of water stress over a large portion of Asia that includes China, India, and Mainland Southeast Asia in a future with unconstrained emissions. We isolate the effects of socioeconomic growth from the effects of climate change in order to identify the primary drivers of stress on water resources. We find that water needs related to socioeconomic changes, which are currently small, are likely to increase considerably in the future, often overshadowing the effect of climate change on levels of water stress. As a result, there is a high risk of severe water stress in densely populated watersheds by 2050, compared to recent history. There is strong evidence to suggest that, in the absence of autonomous adaptation or societal response, a much larger portion of the region’s population will live in water-stressed regions in the near future. Tools and studies such as these can effectively investigate large-scale system sensitivities and can be useful in engaging and informing decision makers.

## 1. Introduction

There is rising concern about the impact of climate change and socioeconomic growth on the future of our water resources, e.g., [1,2]. The global climate system and population as well as the local and global economy determine regional and local water supplies and demands–and these forces can result in complex interactions that require deeper understanding in order to provide actionable information to decision makers for strategic planning in a changing and growing world. An emerging need is evident for modeling tools to capture these complex linkages–especially global-to-local hydro-climatic relationships, managed water systems, and population and economic growth.

Previous literature has included many assessments of the impacts of climatic changes and socioeconomic drivers on water supply and demand ([3,4,5,6,7]; among others). These studies have focused on a limited number of future scenarios, providing valuable insights on the potential changes that may arise from a few plausible futures; however, there is no ability to assess where these courses of events and the subsequent water impacts may lie in terms of a distribution of outcomes–i.e. a risk-based lens to the analyses. Given the complexity of the system, critical questions remain such as:

- For any scenario of future climate, population and economy, can we identify a central tendency as well as the “extreme” outliers (i.e. 5^th^ and 95^th^ percentile)?
- Does any scenario result cluster around a central tendency or mode, and therefore indicate that the outcome is more robust?

Without quantified likelihoods of future outcomes, it is difficult to determine which scenarios should be seriously considered when planning new investments. Here we develop and test an approach to provide regional projections of changes in water supply and demand, and the potential for changes in water stress.

We draw on probabilistic projections of global population, economic growth, emissions and climate developed using an Integrated Global Systems Model (IGSM) developed at MIT [8,9]. Advantages of this approach are (1) likelihoods are explicitly quantified; (2) scenarios are self-consistent, in that a climate scenario drawn from these projections was produced from an emissions scenario driven by an associated population and economic growth scenario (recoverable for our projection of water demands and resource uncertainty); (3) underlying uncertainties in drivers of both economic and earth system response are sampled; (4) cascading uncertainties, as pertaining to climate and economic projections, are properly addressed, while additional uncertain variables increase uncertainty in final outcomes, unless the underlying parametric uncertainties are highly correlated (in which case they have a strong tendency to offset one another).

**Fig 1:**
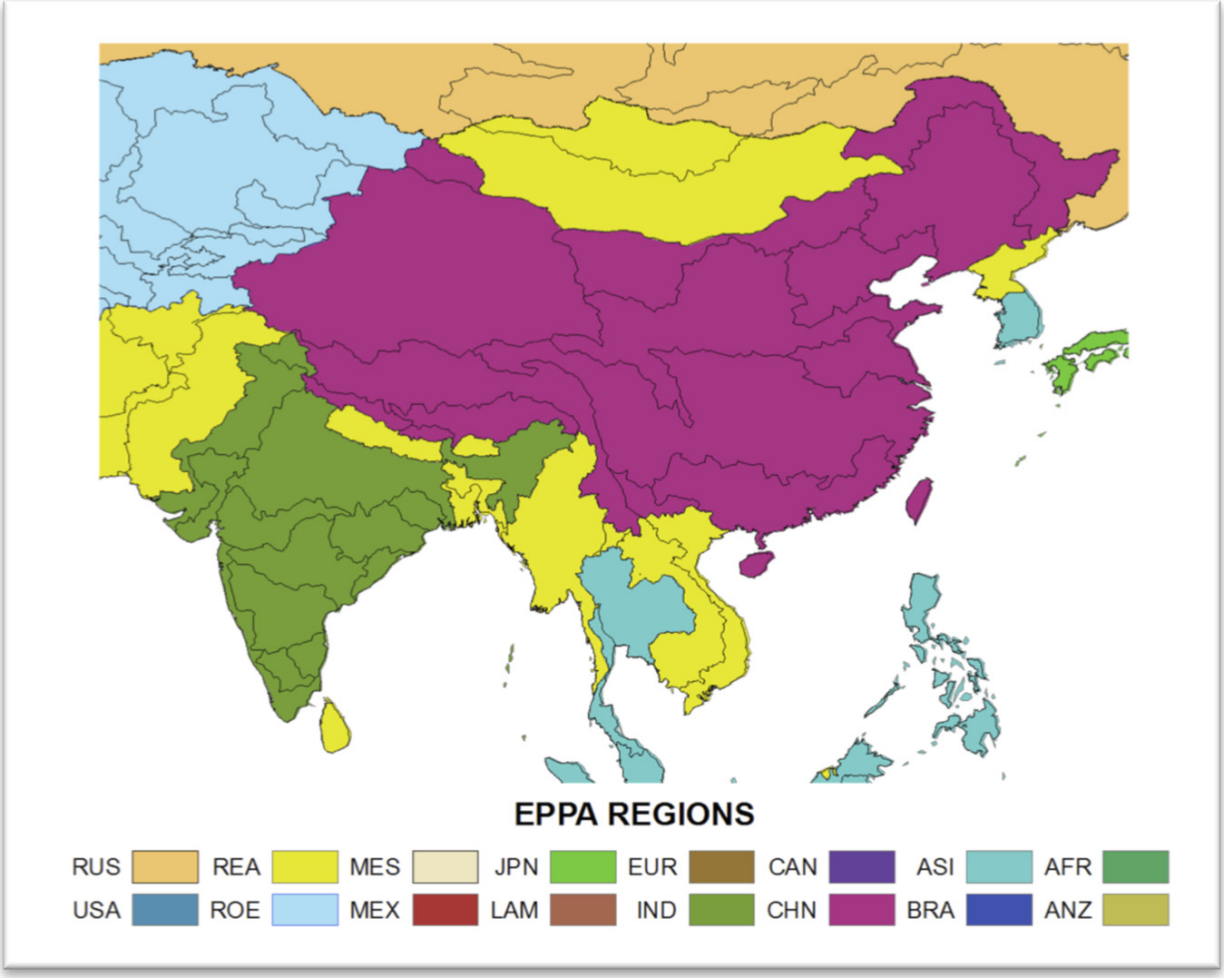
Southeast Asia study region. Black contours delineate Assessment Sub Regions (ASRs) defined for the Water Resource System (WRS) within the IGSM-WRS framework. The color shading indicates the economic regions that are resolved in the Emissions Prediction and Policy Analysis (EPPA) model.

To test the approach we focus on a portion of Asia that includes China, India and Mainland Southeast Asia (Fig 1). This region covers emerging economies constituting almost half of today’s global population, as well as diverse climates that create varied water resource issues involving both surface and ground water. Previous studies in this region have found moderate effects of climate change, some positive and some negative, but raise serious concerns about socioeconomic effects on water-intensive economic sectors [10,11,12,13]. These regional studies, like the previously mentioned global studies, are constrained to a limited number of climatic and socioeconomic scenarios provided by Climate Model Intercomparison Projects (CMIPs) and the Intergovernmental Panel on Climate Change (IPCC).

Our method, in brief, is to apply a Water Resource System (WRS) model developed to work with the IGSM framework [14]. We use 400-member ensembles of climate projections previously developed with the IGSM [8,9] complemented with the pattern-scaling approach of [15] to develop a new 6,800-member ensemble of climate change projections, including variations in the regional pattern of climate change as represented by General Circulation Models (GCMs). The climate projections drive changes in surface water supply through changes in runoff and irrigation demand, and the IGSM economic ensemble projections provide the necessary parameters to estimate changes in water demands for industry and municipal use. A water management module within the WRS allocates, stores and releases water over each year, regulated by a management decision scheme that sets priorities among uses. This allows a distribution of water stress, indicating risks for river basins and sub-basins within our target region of Asia. We project water stress out to 2050. We could apply this same method out to 2100, as other studies have done, when changes in water stress, especially through climate change, are likely to be more substantial; however, for the usefulness of the this study, 2050 will serve as a more applicable planning horizon.

Tools and studies such as these can effectively investigate large-scale system sensitivities and can be useful in engaging and informing decision makers. In addition to demonstrating what results may be derived with a tool such as this, we also hope to highlight the large-scale sensitivities in this specific region—which contains several heavily populated areas already water stressed—with the hope of directing more detailed, project-scale studies within the region.

In Section 2 of this paper, the models and methods are described; in Section 3, the changes in water supply and demand are shown in detail, as well as the resulting water stress risk portfolios; and in Section 4, the main conclusions from this work are presented.

## 2. Models and Methods

### 2.1 The IGSM-WRS and Study Region

**Fig 2:**
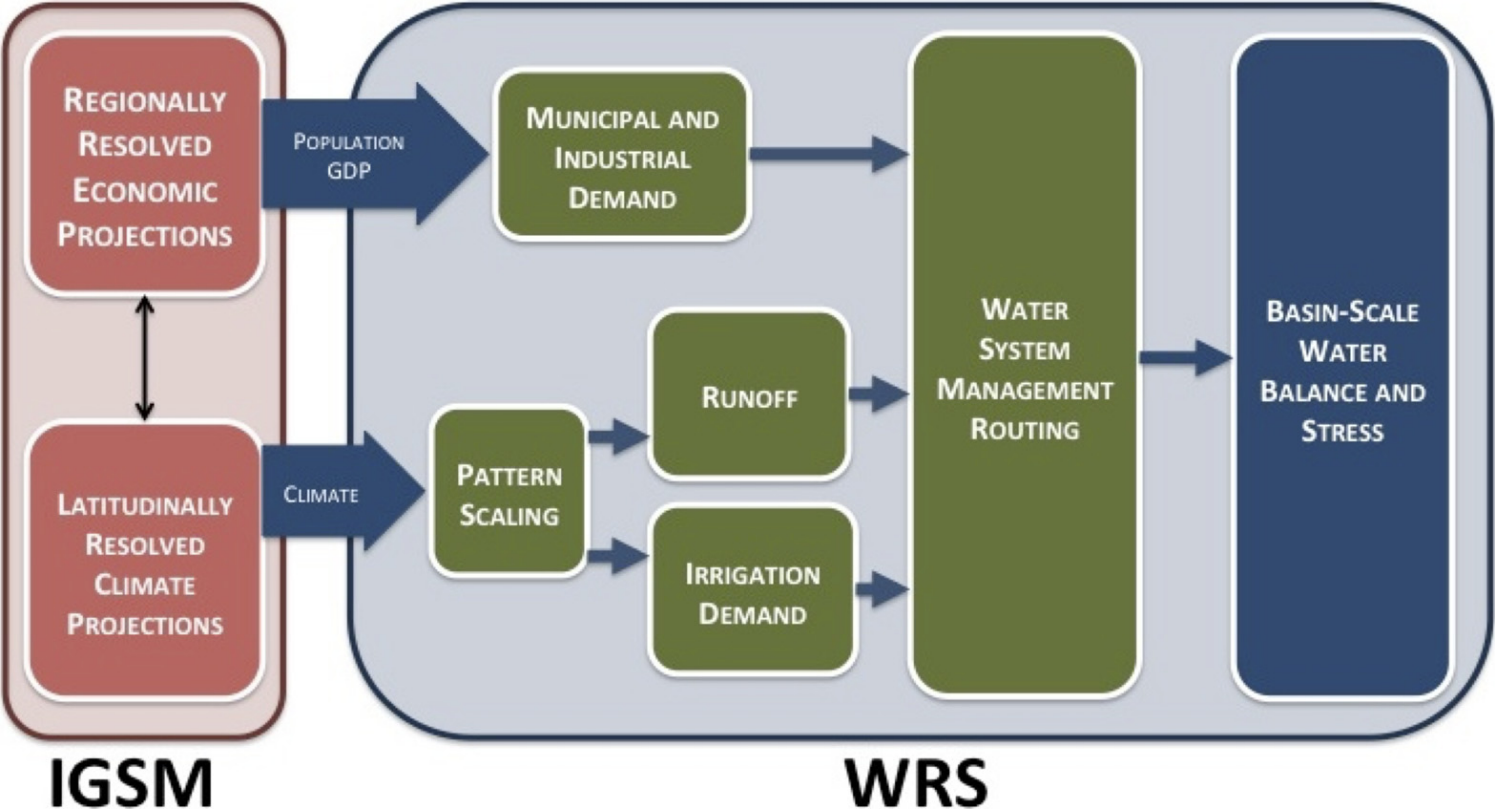
Schematic of connections between components of the IGSM framework and the WRS. Within the IGSM, the EPPA model produces economic projections, calculating population and GDP for each ASR. These determine municipal and industrial demands for water. Climate results from MESM are projected longitudinally via pattern scaling with archived GCM data. CLM determines runoff, and CliCrop calculates irrigation demands. Water demands and surface-water supply are fed into the WSM to optimize the routing of water across all ASRs. The resultant routing is then analyzed via water stress indicators.

Our analysis focuses on the impact of socioeconomic growth and climate changes on the future availability and management of water resources resolved over large watersheds—Assessment Study Regions (ASRs)—across South, Southeast, and East Asia (Fig 1). The basic structure of the WRS as applied here is illustrated in Fig 2, with greater detail provided in [14].

The WRS is driven by economic and climatic projections from the Massachusetts Institute of Technology (MIT) Integrated Global System Model (IGSM) described in [16]. Economic projections are driven by the MIT Economic Projection and Policy Analysis (EPPA) model, a regionally resolved general equilibrium model of world economies (described in [17]), which provides inputs for econometrically estimated relationships of industrial and municipal water requirements based on changes in population and gross domestic product (GDP) [14].

The same EPPA scenarios provide greenhouse gas and other pollutant emissions to the MIT Earth System Model (MESM), which produces latitudinally-resolved climate projections; the IGSM sub-model of atmospheric dynamics and chemistry is 2-dimensional (altitude and latitude) and is coupled to a mixed layer ocean component. As discussed in [9], an ensemble of futures is produced using a Monte-Carlo type framework as well as a Latin Hypercube, which is used to reduce the sample size to 400 members for each ensemble (discussed in more detail in Section 2.2). The parameters sampled to produce the ensembles include 9 related to economics (via EPPA) and 5 related to climate (via MESM). The zonal resolution of the MESM makes it feasible to produce the 400-member ensembles necessary to reasonably resolve the distribution of future climate outcomes [9]. MESM outputs are rescaled to 2° latitude by 2.5° longitude using a pattern-scaling technique [15] based on archived CMIP-3 Climate model simulations. These precipitation and temperature results are used to drive the Community Land Model (CLM) version 3.5 [18] to produce the runoff for each ASR. The CLM, which explicitly represents soil thermal and hydrologic processes, is also implemented within the IGSM as its land surface scheme. The simulated runoff is bias corrected, using a modification of the Maintenance of Variance Extension (MOVE) procedure to ensure that projected flows in each basin are a realistic representation of natural flow conditions, details in [14].

Downscaled precipitation and temperature are also input to the CliCrop [19] component of the WRS, a daily crop water deficit model which projects irrigation requirement. The multiple water demands are inputs to the Water System Management (WSM) component of the WRS that allocates water for consumption and assesses the adequacy of water supplies in light of changing water availability at the ASR level. We use previously published and archived ensemble IGSM runs that consider underlying uncertainty in both climatic (climate sensitivity, ocean uptake and aerosol effects) and economic parameters (labor and energy productivity growth, population, resource availability, technology costs, pollution emissions and substitution elasticities) as described in [9].

For this study, the WRS is configured to represent 54 ASRs over a large region of Asia (see Fig 1). The ASRs are defined by major river basins and parts of river basins contained within a country. For each ASR, available reservoirs are aggregated into a single storage unit that receives water from runoff within the ASR and remaining flows of upstream ASRs. The stored water is allocated to serve human water sector requirements and a required environmental flow. Non-irrigation requirements (for municipal, industrial, and livestock uses) are driven by socioeconomic factors on the assumption they are not significantly influenced by climate; irrigation requirements, on the other hand, are determined by environmental conditions, calculated by CliCrop.

Based on recent evidence over the past decade, global growth in irrigated land area has slowed considerably (e.g., [20,21,22]) even though global food production has steadily increased [23]. This indicates that rising global food demand is being met by increased rainfed agriculture and intensification of existing irrigated land. Given the complexity of interactive socio-economic drivers and environmental pressures, as well as global and national governance that will affect future decisions regarding irrigation expansion (i.e. new dams and reservoirs, e.g., [24]), the irrigated area is held constant (equal to current estimates from FAO and IFPRI) and irrigation efficiencies are also static (see [25]) in these experimental simulations; we focus on whether there is adequate water to meet needs associated with changes in ASR-scale socio-economic activity and climate.

These methods have been described in detail in [14], where the WRS suite of models is compared to observations. As this is an exhaustive exercise, we refer the reader to this study and do not attempt to reproduce it here.

### 2.2 Ensemble Simulations and Scenarios

Our method is to construct numerous ensembles that incorporate the uncertainty in future hydrology and water resources, as affected by uncertainty in climate and economic drivers of water use. We gauge the changes simulated in these ensembles with respect to a single baseline scenario. The baseline scenario represents a 50-year IGSM run with year-2000 water requirements from agriculture, industry, and municipalities and a mean year-2000 climate with 50 years of recent historical inter-annual climate variability. We compare resulting changes in supply, requirement, or water stress with the baseline result to isolate the effect of the long-term mean change in climate. For baseline domestic and industrial water requirements, we use data from [25] that are also used in the global IGSM-WRS, described in detail in [14].

Our projections are designed to distinguish effects on water use of economic and population growth separate from that of future climate change. We create three ensembles of 50-year simulations (2000–2050) of water resource supply and use. In the first, we utilize forecasts of the socioeconomic drivers of water demand to create an ensemble as if only the economy changed (no climate change), which we hereafter refer to as the *Just Growth* ensemble. In the second, we utilize the same economic scenarios and associated emissions, simulating their effect on climate to create another ensemble as if only the climate changed, which we hereafter label the *Just Climate* ensemble. Finally, we develop a large ensemble including both climate change and economic growth, which we hereafter label the *Climate and Growth* ensemble. These ensembles allow us to separately identify the relative importance of climate change and growth, study the combined effect of these changes, and compare them against a baseline ensemble (as if neither climate nor socio-economic drivers changed). The *Just Growth*, *Just Climate*, and *Climate and Growth* ensembles are all generated on the assumption that there are no policy constraints on greenhouse gas emissions. This policy results in a median scenario (of the 400-member ensemble) closest to the A2, used in the AR4, in terms of both GHG concentrations and net radiative forcing, although slightly lower, by 2050. Analyzing mitigation effects, i.e. comparing results with the stabilization ensemble runs, in this region will be handled in a future study.

#### 2.2.1 Baseline Scenario Data

A long-term dataset of near-surface meteorological variables—the Global Meteorological Forcing Dataset (GMFD) [26]—provides the baseline climate in this study. The data is constructed by combining a suite of global observation-based datasets with the National Centers for Environmental Prediction-National Center for Atmospheric Research (NCEP-NCAR) reanalysis. The GMFD data spans the years 1948 to 2008 at the 1° spatial and 3-hourly temporal resolution.

**Fig 3:**
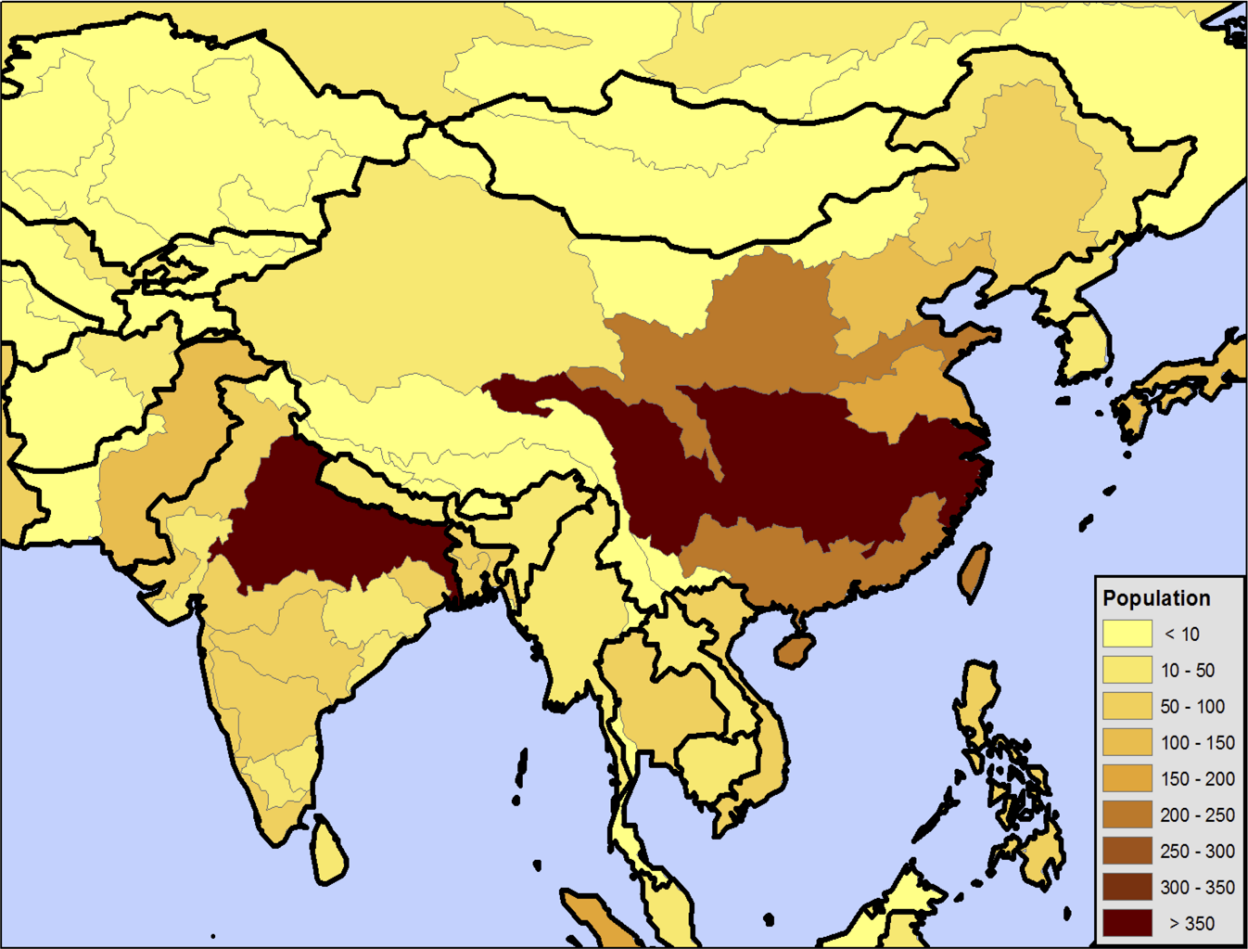
Schematic of the delta method for producing the future climate forcing. The procedure is applied for six near-surface meteorological variables (near-surface air temperature, wind speed and specific humidity at the lowest atmosphere level, total precipitation, shortwave and longwave radiation). The IGSM monthly precipitation is partitioned into 3-hourly based on the observed number of precipitation events within that month and amount of precipitation per each event in the derived 3-hourly baseline precipitation time series.

Six near-surface meteorological variables have been processed: 2 m air temperature, total precipitation, shortwave and longwave radiation, wind speed, and specific humidity. Then, we remove underlying linear trends in the data in order to build a “clean slate” on which to apply future trends. To detrend the 3-hourly forcing, the data is aggregated monthly, then regridded to a 2.5° × 2° resolution. The linear trend is estimated for each of twelve months at each grid over our study region based on the 50-year (1951–2000) period. To bridge the potential gaps between the detrended baseline climate in the last year and the derived future climate (discussed in the following section) at the beginning of the future simulations, for each of the twelve months, we add the 20-year mean (1981–2000)—same years used in developing the future changes discussed in Section 2.2.2—to the monthly residual across the 50-year, then calculate the ratio of this sum to the aggregated monthly time series. The detrended 3-hourly data at each grid are then obtained by scaling the original 3-hourly time series with this monthly ratio. Note that the same ratio is applied across each of the 3-hourly time steps within a specific month. The resulting climate, which is detrended and beginning at the year 2000 mean is our baseline scenario run through the WRS suite of models as discussed in Section 2.1. This procedure is mapped in Fig 3.

#### 2.2.2 Climate Change and Growth Scenario Data

**Fig 4:**
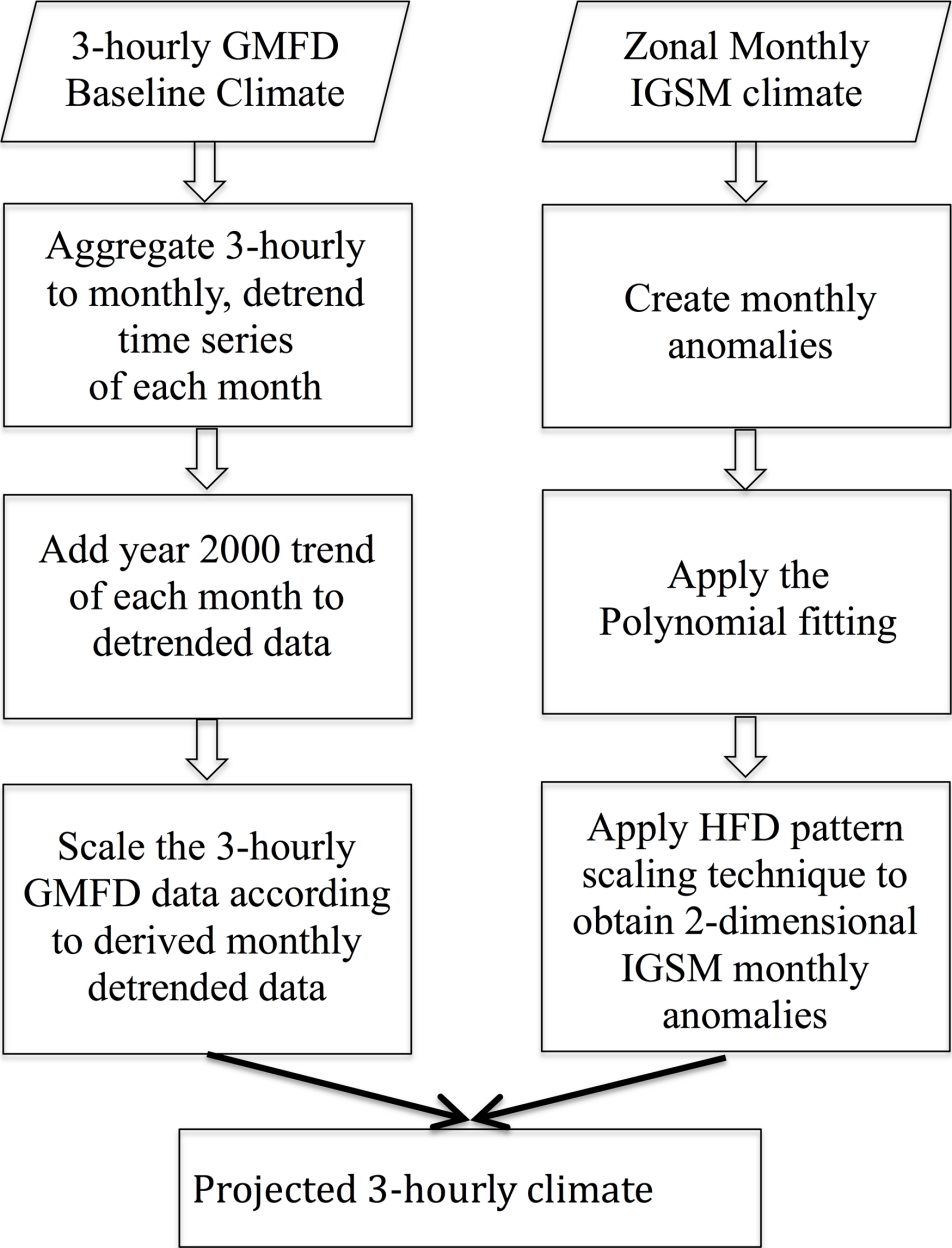
Year 2000 global distribution of population (in millions). Population is projected onto the Assessment Sub Regions (ASRs) of the WRS (Water Resource System) water-management network of river basins. Black contours denote political boundaries.

This study considers changes in GDP and population obtained in the unconstrained emissions (UCE) ensemble analyzed by [16]. The UCE policy uses the global ensemble of population projections described in [27]. To be consistent with the IGSM uncertainty formulation, socioeconomic projections are provided by EPPA region (Fig 2). To provide these population projections at the ASR scale, the EPPA regions’ rate of population changes are mapped to the ASR regions within each EPPA region, following the technique used in Strzepek *et al*. (2013). ASR-based population projections use the growth rates from EPPA, with the current populations at the ASR level developed by IPFRI [25] (see Fig 4).

The MIT IGSM is designed to quantify various sources of uncertainty in climate projections. The fully coupled IGSM is forced from 1861 to 1990 by observed changes in greenhouse gas concentrations, and from 1991 to 2100 by emissions of greenhouse gas and aerosol precursors projected by the EPPA model [8]. Our 400-member climate forecast ensemble was conducted based on different value combinations of three key climate parameters: effective climate sensitivity, ocean heat uptake rate, and net aerosol forcing [8]. The value of each parameter is sampled from its probability distribution obtained by comparing the twentieth-century simulations with observations of surface, upper-air, and deep-ocean temperature changes [28]. The climate forecast ensemble is calculated for each of five emission pathways: Unconstrained Emissions (*No Policy*) and four greenhouse-gas stabilization levels (*Level 1*, *Level 2*, etc.). In this study, only the results for Unconstrained Emissions are presented.

In the assessment of regional climate change impact on water resource management in Southeast Asia, we use a simple downscaling method, or delta method [29], to construct a series of atmospheric forcing to conduct our ensemble simulations. The method is based on applying the interpolated monotonic changes in climate from the IGSM projections to the baseline climate, accounting for any bias (or trend) in the baseline climate under future climate change. This method assumes that changes in climates (i.e. multi-year anomalies) are most relevant in the IGSM projections, and that the relationships between variables in the baseline climate–including periodic and irregular fluctuations in variables–are likely to be maintained. The zonal anomalies (delta) are derived for the IGSM monthly time series of 2001 to 2100 with respect to the 20-year (1981–2000) climatology for each meteorological variable and each of the 400 climate forecast scenarios. There exist some biases in the IGSM-simulated zonal precipitation of potential climate change as compared to the CMIP-3 GCM projections, and we correct such biases based on the monthly zonal precipitation climatology of three periods (2011–2040, 2041–2070, 2071–2100) from the SRESA2 simulation of the Intergovernmental Panel on Climate Change (IPCC) 4^th^ Assessment Report (AR4) [30]. The monthly zonal precipitation climatology from each of the 17 GCMs in the SRESA2 scenario has been analyzed to examine the impact of model structure in bias correction.

To account for the uncertainty in regional climate change, a downscaling technique [15] is employed to expand the IGSM monthly zonal anomalies of precipitation and 2 m air temperature of each of 400 climate forecast scenarios across longitude at 2.5° × 2° by applying longitudinally-resolved patterns, from observations and from climate model projections archived for the IPCC AR4. The observed patterns for precipitation and temperature are derived from the 31-year (1979–2009) monthly GPCP v2.1 data set [31] and the 20-year (1981–2000) monthly Princeton data set, respectively. The pattern shifts in response to human-forced change are derived based on the same 17 GCM simulations from the IPCC AR4 SRESA2 emission scenario. The resulting meta-ensemble (400 × 17 = 6,800 members) of the 2.5° × 2° IGSM monthly anomalies (precipitation and temperature) is used for the Gaussian quadrature procedure presented later.

The IGSM monthly zonal anomalies of each climate forecast scenario are further interpolated using a polynomial of degree 3, with a least-squares fit, to produce a smooth time series (removing rapid changes in gradient in the vicinity of the data points). This is performed for all the near-surface meteorological variables, except that the zonal precipitation anomalies go through the additional bias correction (as described before) prior to the interpolation procedure. A similar downscaling technique [15] is used to map the interpolated IGSM monthly climate across longitude. These anomalies are then added to the detrended 3-hourly baseline climate to construct the future 3-hourly atmospheric forcing (so called “delta method”), which is used to drive the CLM offline from 2001 to 2050 to simulate the runoff.

The combined effect of growth and climate are then explored in combination through WSM. The IGSM-WRS is integrated to 2050 for all cases. The following analyses will focus on the ability of the ASRs to meet water demands [14] and the relative stress that these demands place on renewable surface water and water available within the managed system.

### 2.3 Ensemble Thinning via Gaussian Quadrature Procedure

Due to computational limitations, running the full ensemble of 6,800 members is infeasible. For this reason, we use a Gaussian Quadrature approach, as described in [32], to produce a subset and respective weights that represent the full ensemble. The Gaussian Quadrature approach identifies a set of indices for the ensemble members, and then identifies a subsample of simulations for which the values of the identified indices are distributed similarly to that of the full ensemble. Thus, we select a series of indices—or summary statistics—that characterize relevant differences among the ensemble members. The number of statistics used determines the size of the resulting subset (i.e., more statistics results in a larger subset) related to the number of equations to solve in order to obtain the Gaussian Quadrature (originally proven in [33]), with more detail for the specific application in [33].

Two key impacts on water resources are runoff and irrigation demand. These impacts integrate many aspects of different climate scenarios including precipitation and Potential Evapotranspiration (PET). [34] developed the Climate Moisture Index (CMI), which uses the ratio of annual precipitation (*P*) to annual PET as follows:

$$CMI=\left(\frac{P}{PET}\right)-1\;when\;P<PET$$

$$CMI=1-\left(\frac{PET}{P}\right)\;when\;P\geq PET$$

CMI may range from +1 to -1, with wet climates showing positive CMI, and dry climates negative CMI. [35] demonstrated that changes in CMI are highly correlated with changes in runoff and irrigation demand. Thus, CMI is a single, simple to calculate index that is highly correlated with major impacts of interest in this study.

**Fig 5:**
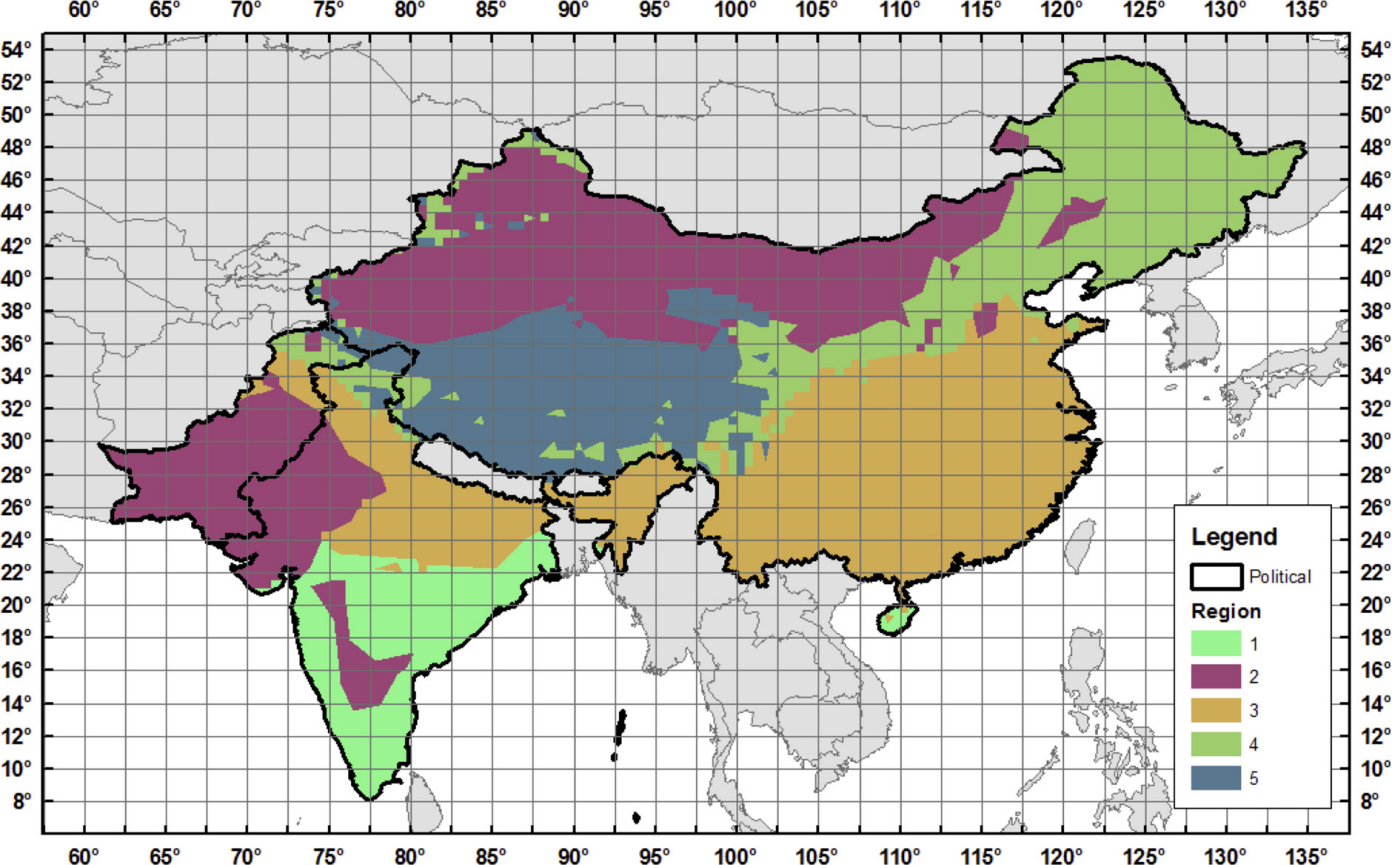
Regions used in the Gaussian Quadrature summary statistics with 2.5° longitude by 2.0° latitude HFD grids. Colored polygons denote the 5 regions used for the Gaussian Quadrature thinning, based on the Koeppen-Geyger Climatic Zones. Black lines are the political boundaries of China, India, and Pakistan.

We calculated CMI for each of the 6,800 climates for 5 regions based on the Koeppen-Geyger climatic zones (shown in Fig 5) and for two 10-year time slices: a mean over 2025 to 2034 and a mean over 2041 to 2050, in order to match the time slices used in the Results discussed later. In the CMI calculation, we use the modified Hargreaves equation to calculate PET [36], which uses a relationship between precipitation and temperature. Since WRS also accounts for changes to GDP and population, we add four more indices: year 2050 GDP and population for both India and China. This leaves us with 14 indices total: 10 for climate (5 regions over 2 time slices) and 4 for socioeconomics.

**Fig 6:**
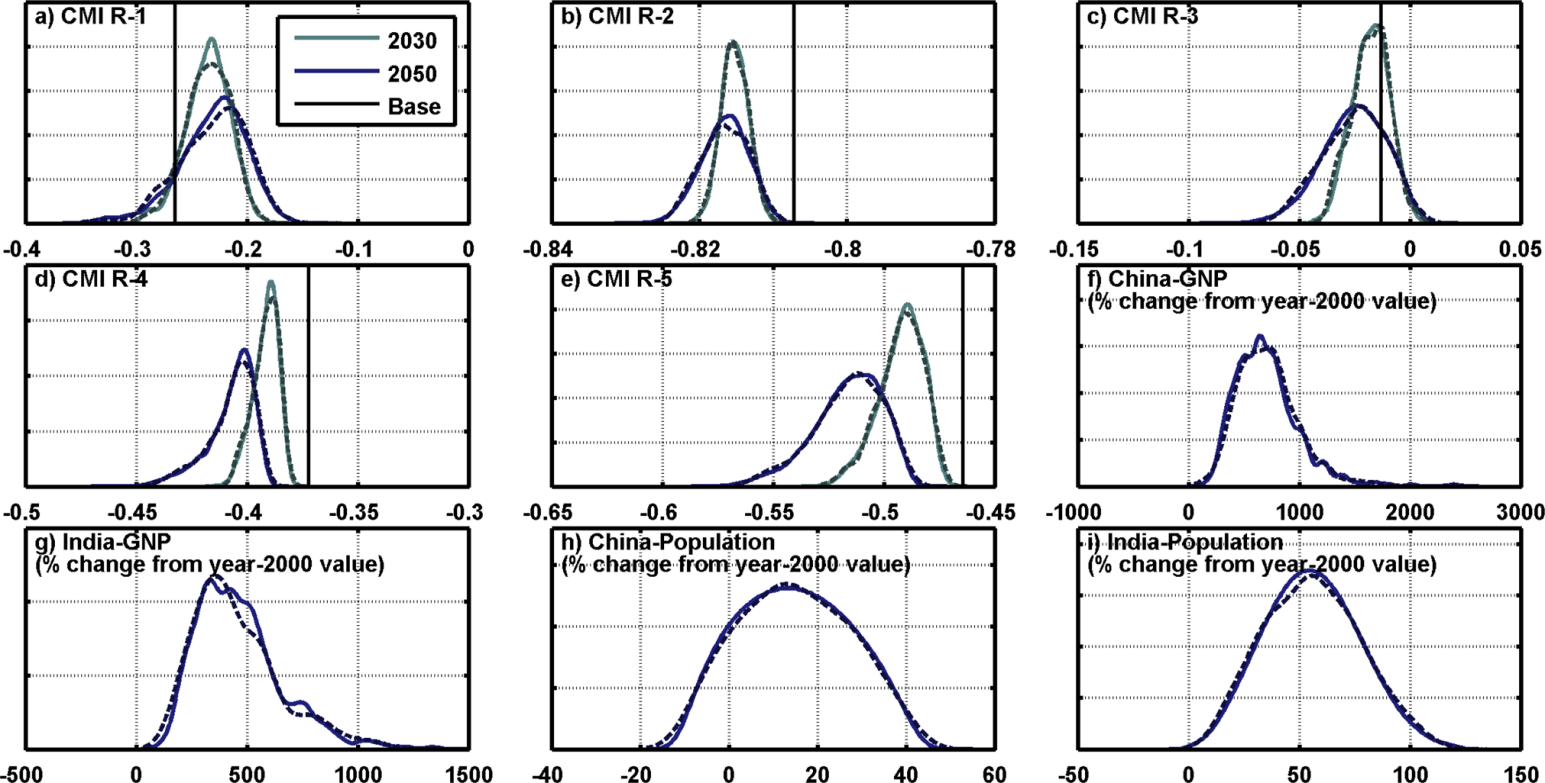
Distribution of the 14 Climate Moisture Index (CMI) statistics used in the Gaussian Quadrature thinning procedure. The solid lines show the values for the full 6,800-member ensemble. Dashed lines are the Gaussian Quadrature subset distributions for comparison. The solid vertical lines on the CMI plots (a to e) show the baseline CMI values.

Fig 6 shows the distribution of the 14 variables for the full ensemble. In this figure, plots a) through e) show the CMI of the 5 regions for the two time slices. In these CMI plots, the dark black line marks the base CMI value. Plots f) and g) show GDP for China and India in 2050 as a percent change from the year-2000 value, and plots h) and i) show population for China and India in 2050, also as a percent change from the year-2000 value. The distributions of the resulting subset are shown as dashed lines. As shown, the Gaussian Quadrature procedure successfully reproduces the original 6,800-member ensemble with a sub-sampled set of 551 members. We then use this sub-sampled ensemble to perform our water resource assessment.

### 2.4 Measures of Water Stress

We use two measures of stress to understand the impacts of changes in climate and growth. The first measure, Unmet Water Requirement (UWR), is the percentage of the total water requirement that is not met by the system. UWR is the main component of the objective function in WSM and is a direct aggregate measure of water stress in each ASR. We calculate UWR as follows:

$$UWR=\left(1-\frac{total\;water\;consumption}{total\;water\;requirement}\right)\times 100\%$$

**Fig 7:**
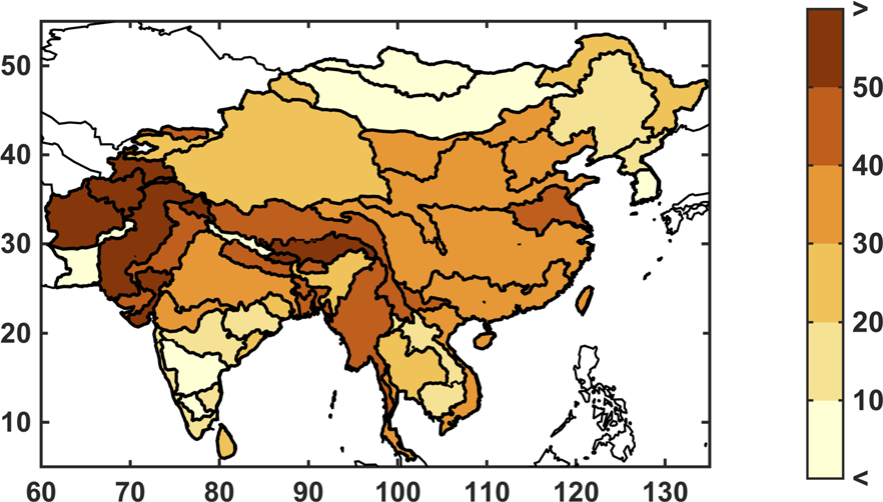
Baseline unmet water requirement (%) for the study region at the ASR level. Unmet requirement is defined as total consumptive use divided by the total water requirement.

In the global WRS model, total water requirement is an estimate of the amount of water that would be consumed given socio-economic factors, climate conditions, and current infrastructure, if water were an unlimited resource. For example, if the total water requirement—irrigation, industrial, and municipal—is 100 billion cubic meters (BCM) and the system can only deliver 90 BCM, the UWR would be 10%. A UWR of 0% indicates that all crops (as well as the other water requirement sectors) are without water stress. The WSM module allocates domestic and industrial consumption requirements to be satisfied first (given sufficient water supply) and the agriculture sector must absorb the loss. Since irrigation is by far the highest requirement for water, it is extremely rare that domestic and industrial sectors absorb any loss from the water limitations. Furthermore, since crops are irrigated depending on their value and water availability, many crops are partially irrigated on a regular basis, which is why we see unmet requirement in the baseline scenario (see Fig 7). Partial irrigation complicates the interpretation of UWR in the baseline scenario, so we focus on changes in UWR, assuming that these changes indicate additional stress in a given region. For instance, an increase in unmet requirement would likely decrease the supply from the agriculture sector, which could increase food prices.

The second measure, a Water Stress Index (WSI), is used to assess the stress on the water resource system for each ASR. For this, we use the metrics adopted for other applications of the IGSM-WRS [14,37]. Our WSI, similar to that developed by [38] is based on input water flows (from surface runoff and upstream ASRs) and desired withdrawals, as a measure of the pressure that human water uses exert on renewable surface fresh water. This measure does not calculate unmet requirement; instead, it gauges stress on the natural water system through its accounting for withdrawal and consumptive uses. WSI is calculated as the ratio of each ASR’s mean annual total withdrawal (*TW*), which by definition includes consumptive loss, to the mean annual runoff (*RUN*) generated within the ASR, plus inflow (*INF*) from any upstream ASR that flows directly into it, as described by [14]:

$$WSI=\frac{TW}{RUN+INF}$$

**Fig 8:**
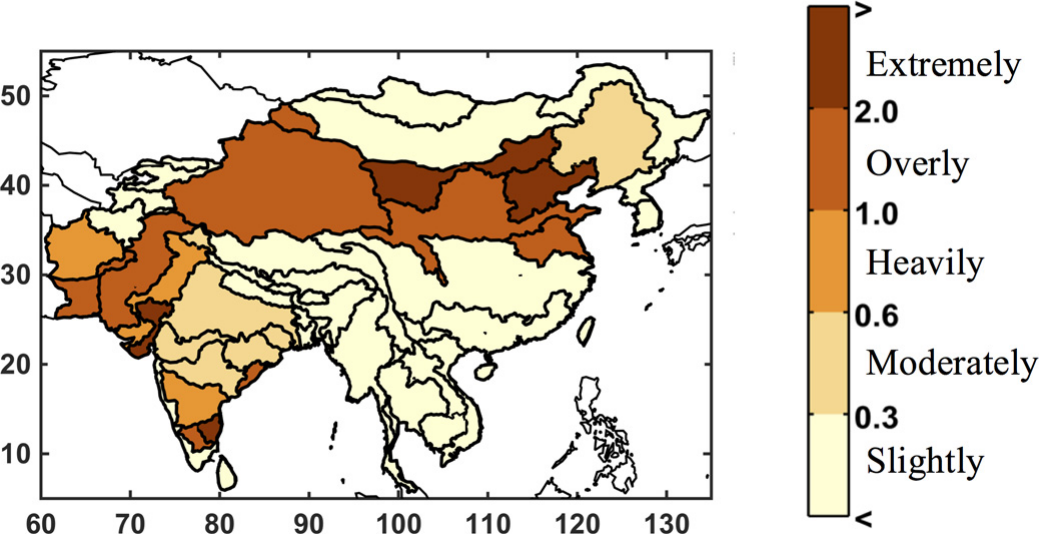
Distribution of water stress index by ASR, as simulated by IGSM-WRS from the baseline climate run.

For only the municipal and industrial sectors, water requirements included in *TW* are represented by consumptive use in the model.—with additional consideration for reuse within the basin to assess total withdrawal. To estimate withdrawal, we use common ratios that represent the fraction of consumption over withdrawal. Inflow to any given ASR is a consequence of flow regulated from upstream ASRs; therefore WSI is an evaluation metric of the managed water system as simulated by WRS. Irrigation receives its total withdrawal, with its return flow credited to the downstream ASR (see [14] for details). We characterize the severity of water stress according to [38], which classifies an ASR’s water use as slightly exploited when WSI < 0.3; moderately exploited when 0.3 ≤ WSI ≤ 0.6; heavily exploited when 0.6 ≤ WSI ≤ 1; overly exploited when 1 *≤* WSI < 2; and extremely exploited when WSI ≥ 2. Similar water-stress indices are computed in other studies and generally consider a threshold of 0.4 to indicate severe water limitation [7]. Fig 8 shows the WSI for the baseline scenario. As shown, a large portion of northern China as well as India and the Indus River systems experience at least moderate to extremely exploited water conditions.

## 3. Results

### 3.1 Distributional Changes in Climate Parameters

**Fig 9:**
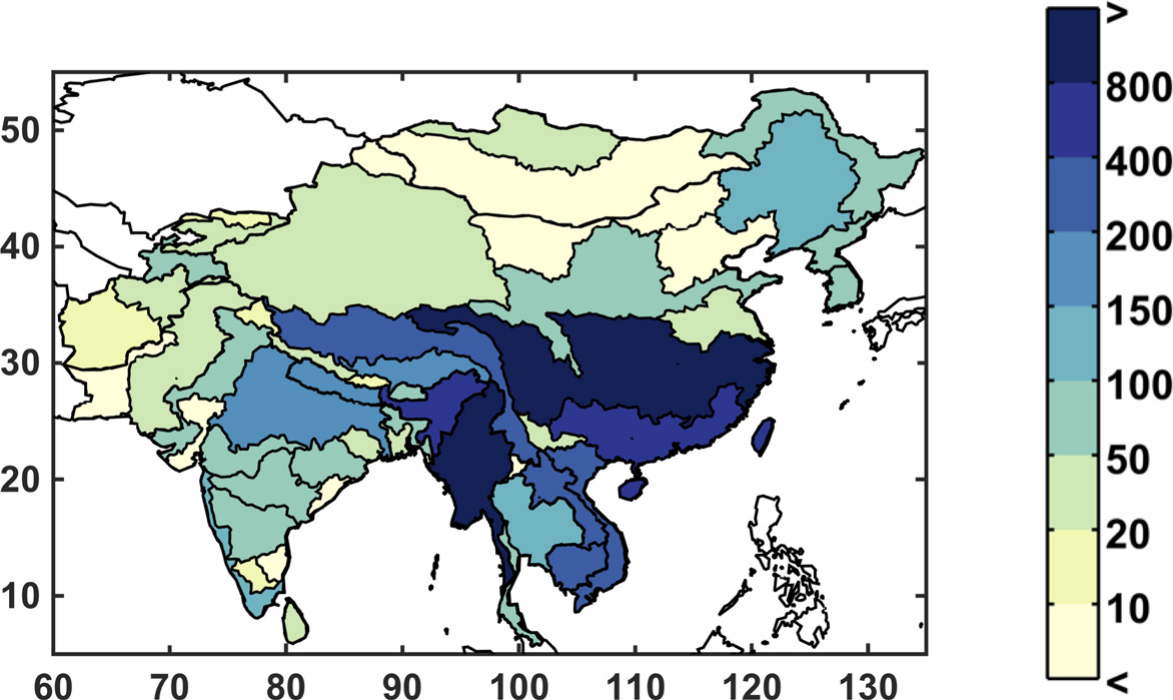
Baseline annual runoff by ASR (in billion cubic meters per year).

In the IGSM-WRS framework, two variables respond to changes in climate: runoff, which provides surface water supply to the ASR; and irrigation requirement, which is an estimation of farming water requirements. The baseline runoff is shown in Fig 9 in billion cubic meters (BCM). In general, there is substantial runoff in the southeast, which benefits from a wet and humid climate, while the north and far west of the region are especially dry. Note that, in order to keep units consistent, runoff is not normalized by area, so larger ASRs have more runoff in part due to the contributing land area.

**Fig 10:**
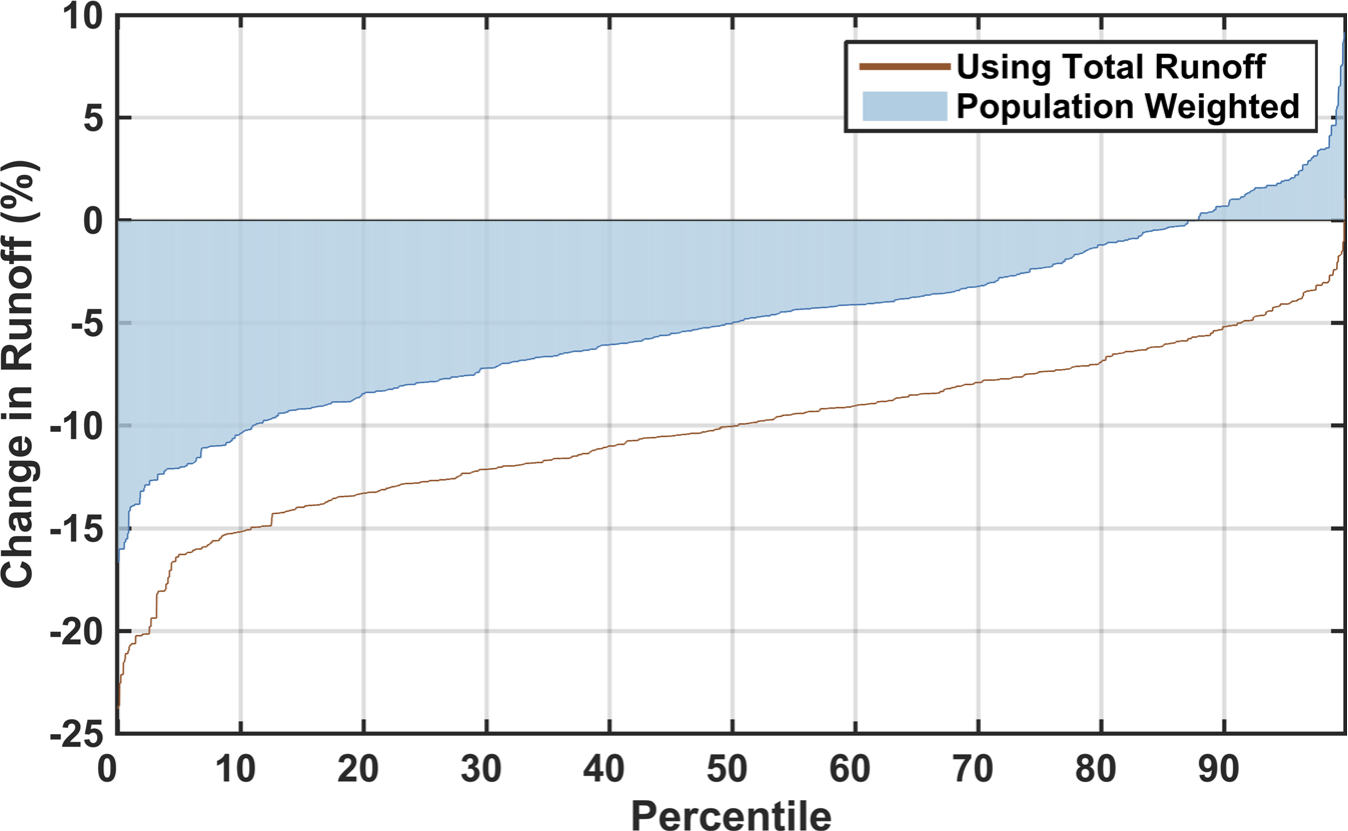
Percentage change in runoff across all ensemble members. Each point in the line represents one of the 551 members with appropriate weights from the Gaussian Quadrature (Section 2.3). The percent change in runoff represents a weighted-averaged result for the entire domain of study region (Fig 1)—such that for every member's result in the distribution shown, each ASR's runoff has been weighted by its population (Fig 3).

We take two approaches to present the large number of future runoff changes: (1) we show example maps of probability points on the distribution of a single metric, maintaining the geographic spatio-temporal patterns in each scenario, and (2) we simplify complex results by ignoring spatio-temporal correlations and mapping points for specific values in the ASR probability distributions. For (1), first we characterize the resulting runoff of each scenario using a single metric across area and time. We find a strong likelihood that runoff will decrease for the majority of the population (Fig 10). The values shown (as a cumulative probability distribution) are calculated using a population-weighted mean of the percent change in annual runoff from all the ASRs by 2050, with ensemble members sorted from driest to wettest. Around 87% of scenarios suggest an overall decrease in runoff. While the scenarios shown in Fig 10 indicate a predominate tendency toward a relative decrease in runoff *averaged for the entire region*, we find that the variability across ASRs is quite diverse.

**Fig 11:**
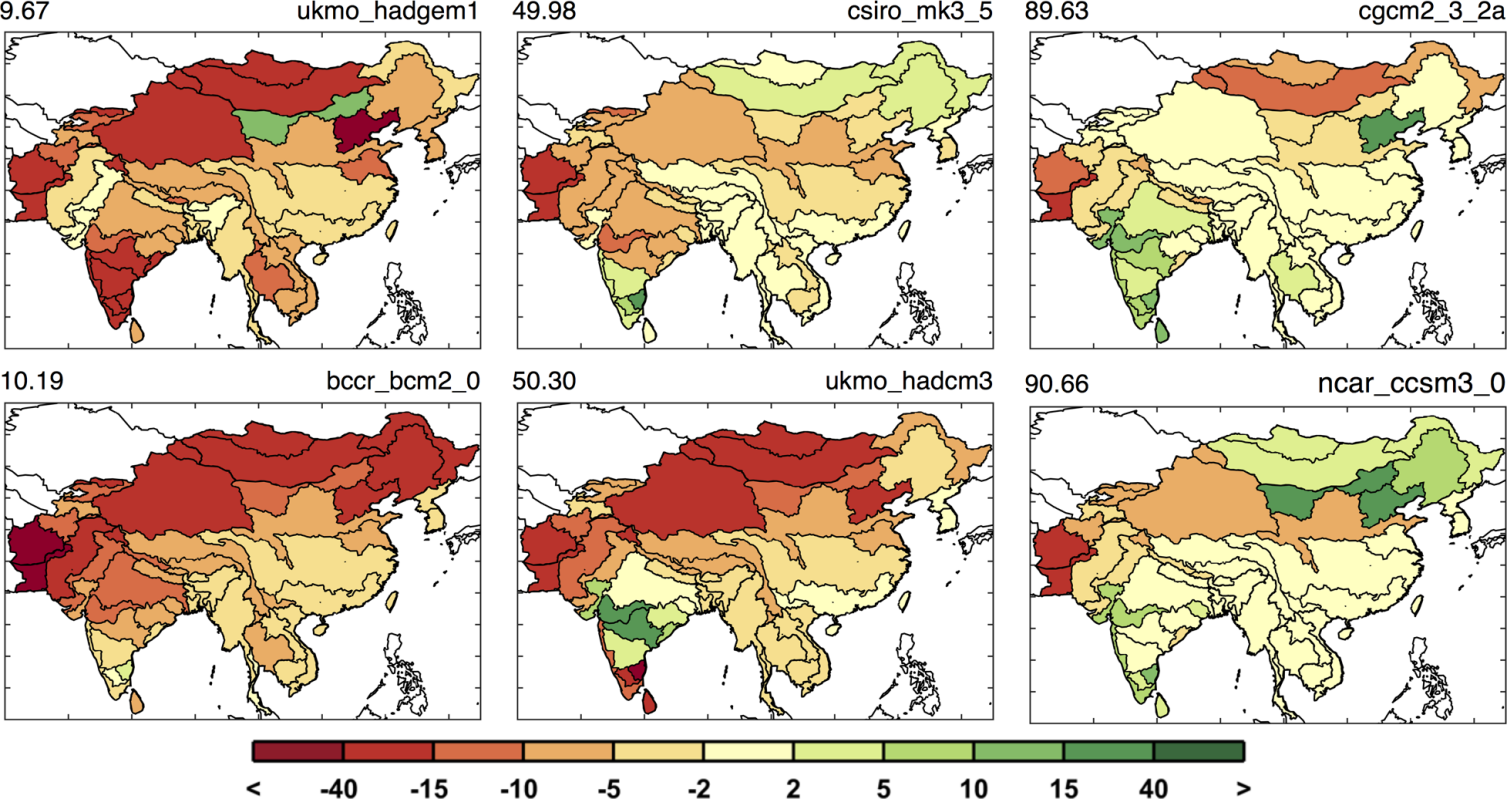
Runoff change patterns (in %) around the 10^th^, 50^th^, and 90^th^ percentile. Two are shown for each percentile, based on the mean runoff change for the region (the metric used in Fig 10). Top label shows the percentile (left) and the GCM name (right.)

**Fig 12:**
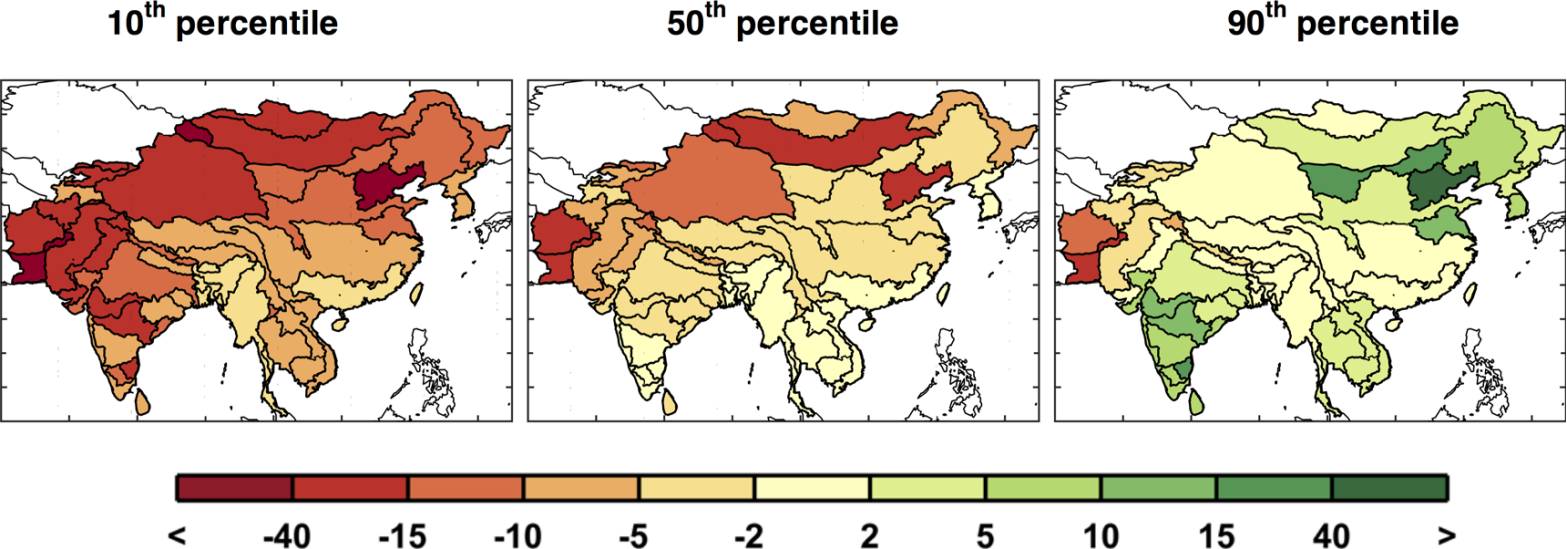
Changes in ASR runoff (%) calculated point-wise by ASR. These are changes in decadal averaged ASR runoff from the baseline to the future scenarios averaged over 2041–2050 for the 10^th^, 50^th^, and 90^th^ percentiles.

Fig 11 highlights this situation. The first column shows results around the 10^th^ percentile, the second column around the median, and the third column around the 90^th^ percentile. With these we can see there are patterns that persist in most cases, e.g., reduced runoff in western Pakistan and Afghanistan, but many of the ASRs provide varying results depending on the specific climate pattern. The diversity in the regional patterns of runoff change is further illustrated by mapping the 10^th^, median, and 90^th^ percentiles of runoff change for each ASR in a “point-wise” fashion (Fig 12). As a result, these maps display a general inference about the runoff change distribution at each ASR, but do not represent the likelihood of a specific climate pattern. In this context, for any given ASR, a drier climate would be anticipated for those in the north and west portions and little to no change in the south, as compared to the baseline scenario. Most regions have scenarios that project wetter and others that project a drier future but there are exceptions. Afghanistan and Pakistan are especially prone to a drier future climate, as 90% of the scenarios indicate decreased runoff, and southern and western China are more likely to decrease or remain the same, as even in the 90^th^ percentile a wetter climate is not predicted.

**Fig 13:**
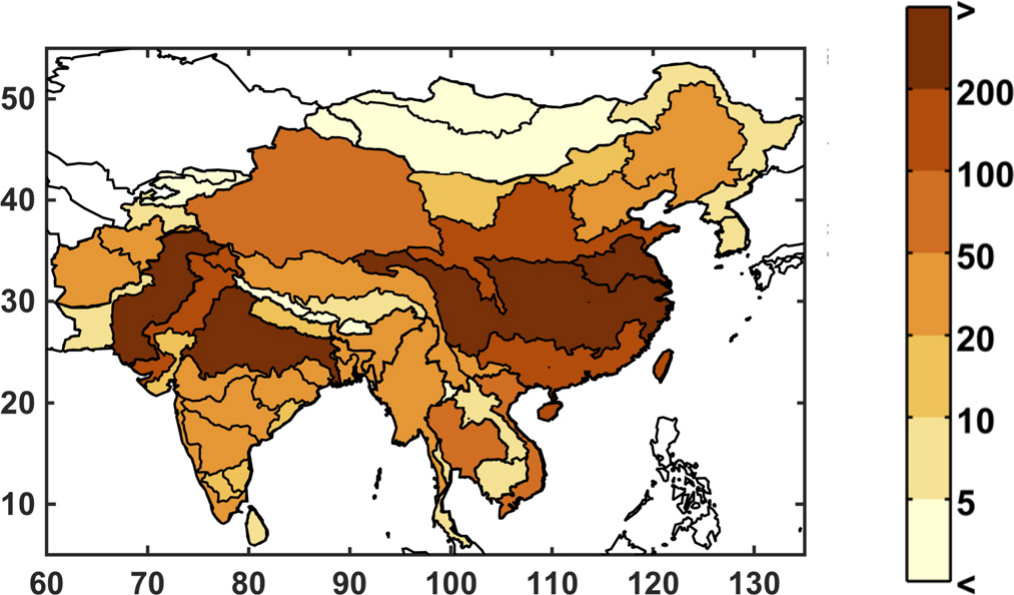
Baseline irrigation requirement (in billion cubic meters)

**Fig 14:**
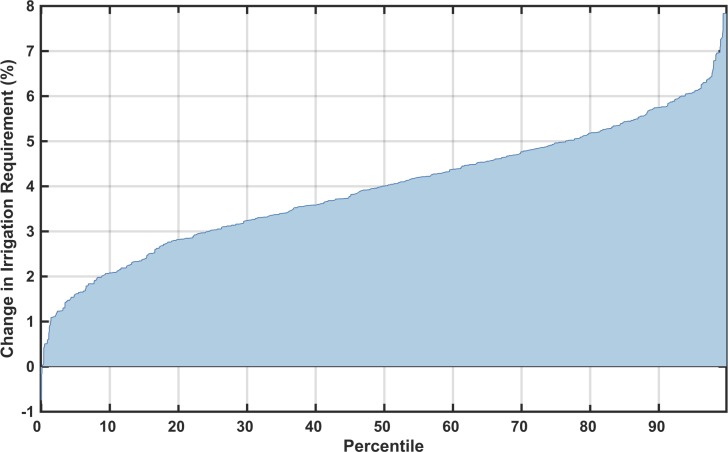
As in Fig 9, but shown for percentage change in irrigation requirement across all ensemble members. Each point in the line represents one of 551 climate scenarios.

The other calculated metric within the WRS framework that is influenced by the IGSM's climate response and pattern-scaling is the irrigation requirement—an estimate of the amount of water that farming in an ASR would use if there were an abundant water supply (given irrigated area per crop and irrigation efficiencies). In this modeling framework, irrigation requirement responds to changes in precipitation and temperature, rising when soil conditions are drier and falling when they are wetter, without exceeding the maximum water needed by the crop. Baseline irrigation requirement is shown in Fig 13. We calculate the percentage change in irrigation requirement, weighted by population, for each scenario. A distributional summary across the ensemble members is shown in Fig 14, ordered from least to greatest. All of the simulations result in increased irrigation requirement across the region, with the least at around no change and the most around 8%.

**Fig 15:**
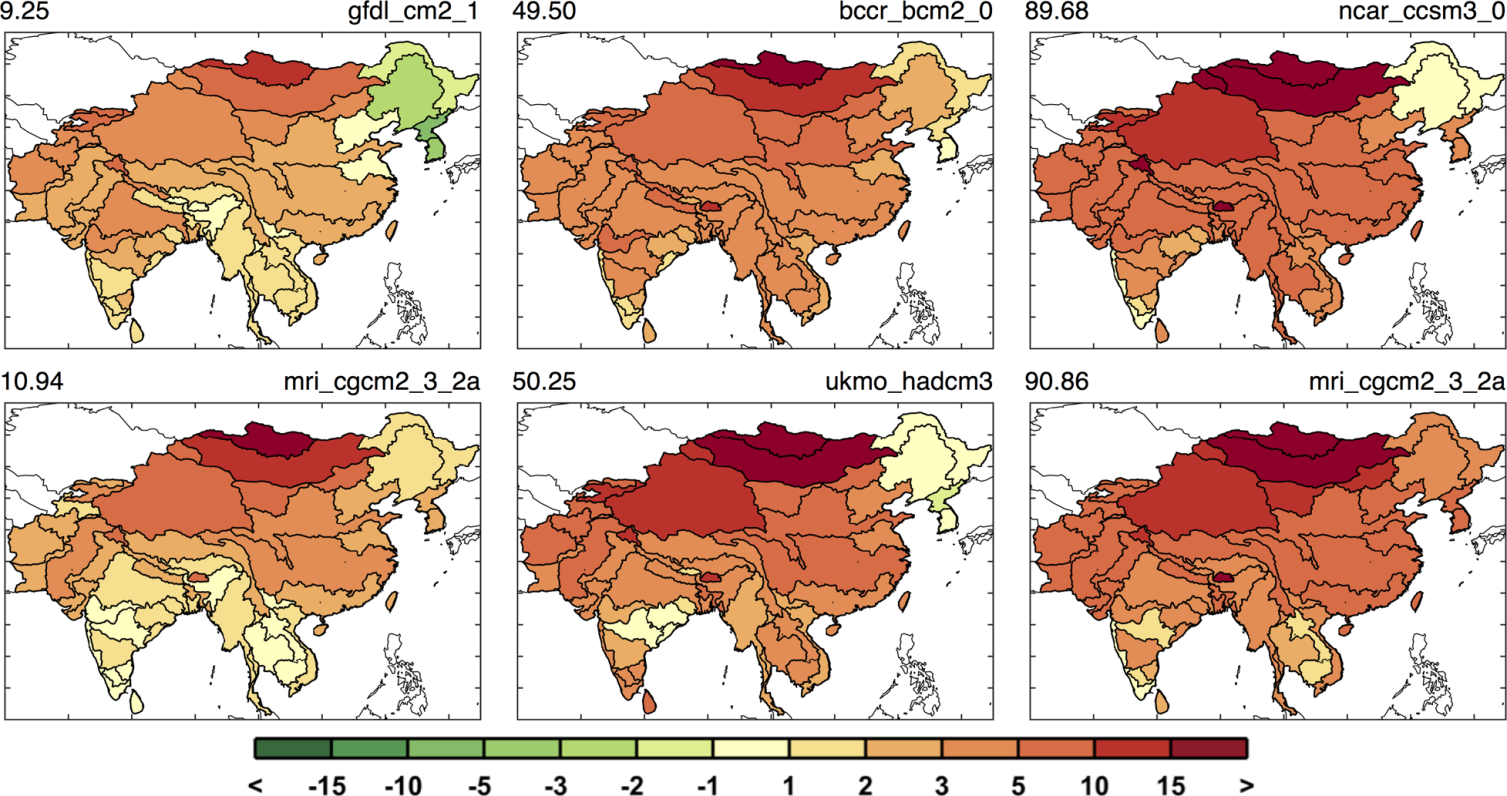
Irrigation requirement change patterns (in %) around the 10^th^, 50^th^, and 90^th^ percentile, two each based on the mean irrigation requirement change for the region. Top label shows the percentile (left) and GCM name (right.)

Similar to Fig 11, in Fig 15 we show examples of six maps of changes in irrigation requirement: two from ensemble members near the 10^th^ percentile, two near the 50^th^ percentile, and two near the 90^th^ percentile results. Again, we see that different climate patterns can result in a similar value of the metric used in Fig 14. In these examples, we do generally see more drying in the north and west and less drying in the south, although not all examples shown adhere to these general patterns.

**Fig 16:**
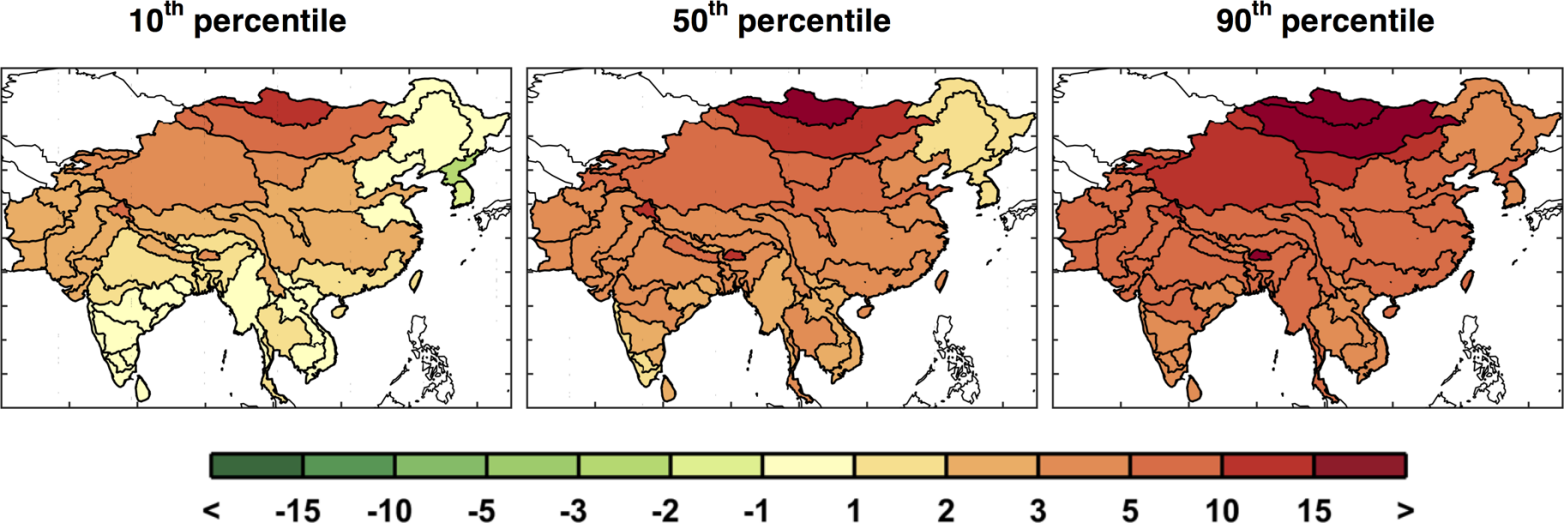
Changes from baseline in irrigation requirement (%) calculated point-wise by ASR, showing changes in decadal averaged ASR irrigation requirement from the baseline to the future scenarios averaged over 2041–2050 for the 10^th^, 50^th^, and 90^th^ percentiles.

Similar to the point-wise maps of runoff shown in Fig 12, individual ASR changes in irrigation requirement are mapped in Fig 16. Since precipitation and temperature are the main drivers for both runoff and irrigation requirement estimations, we see a similar pattern in both maps, with the north drier (i.e., increased irrigation requirement) than the south although both are indicating that irrigation is more likely to increase. Since the irrigation sector is by far the largest requirement for water in this region, small changes in mean irrigation requirement can have a substantial impact on the water sector within each ASR.

### 3.3 Distributional Changes in Growth Parameters

Domestic and industrial water requirements are both driven by changes in growth in the WRS framework. Although these consumptive requirements are smaller than the irrigation requirements in the baseline scenario, in most ASRs they do play a significant role in the future scenarios, depending on population growth and GDP projections.

#### 3.3.1 Domestic Water Requirements

**Fig 17:**
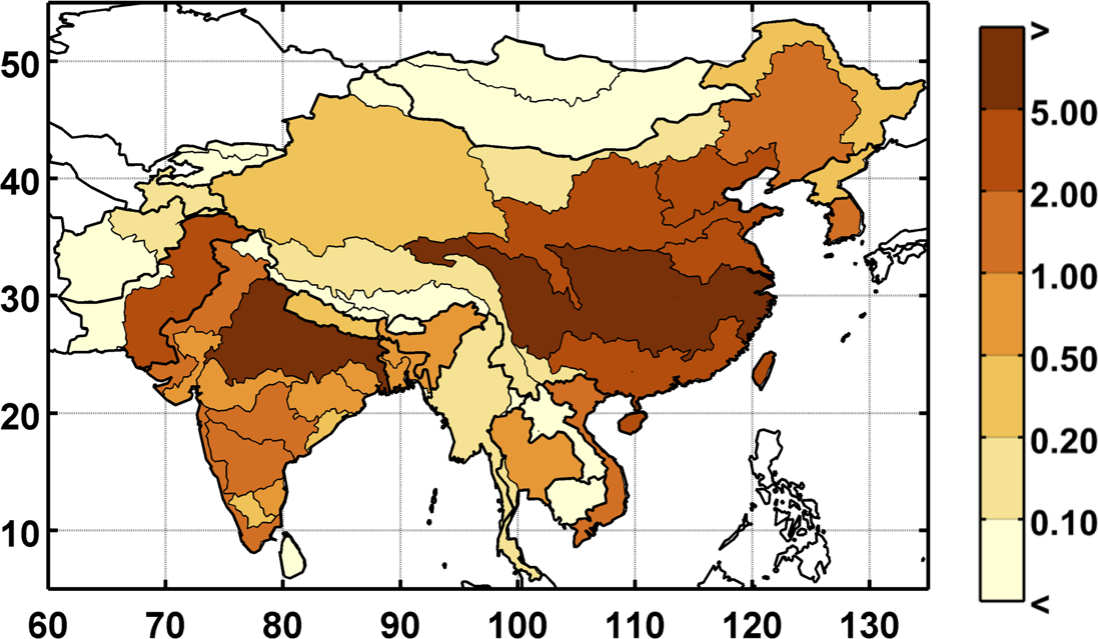
Baseline domestic water requirement (in billion cubic meters).

**Fig 18:**
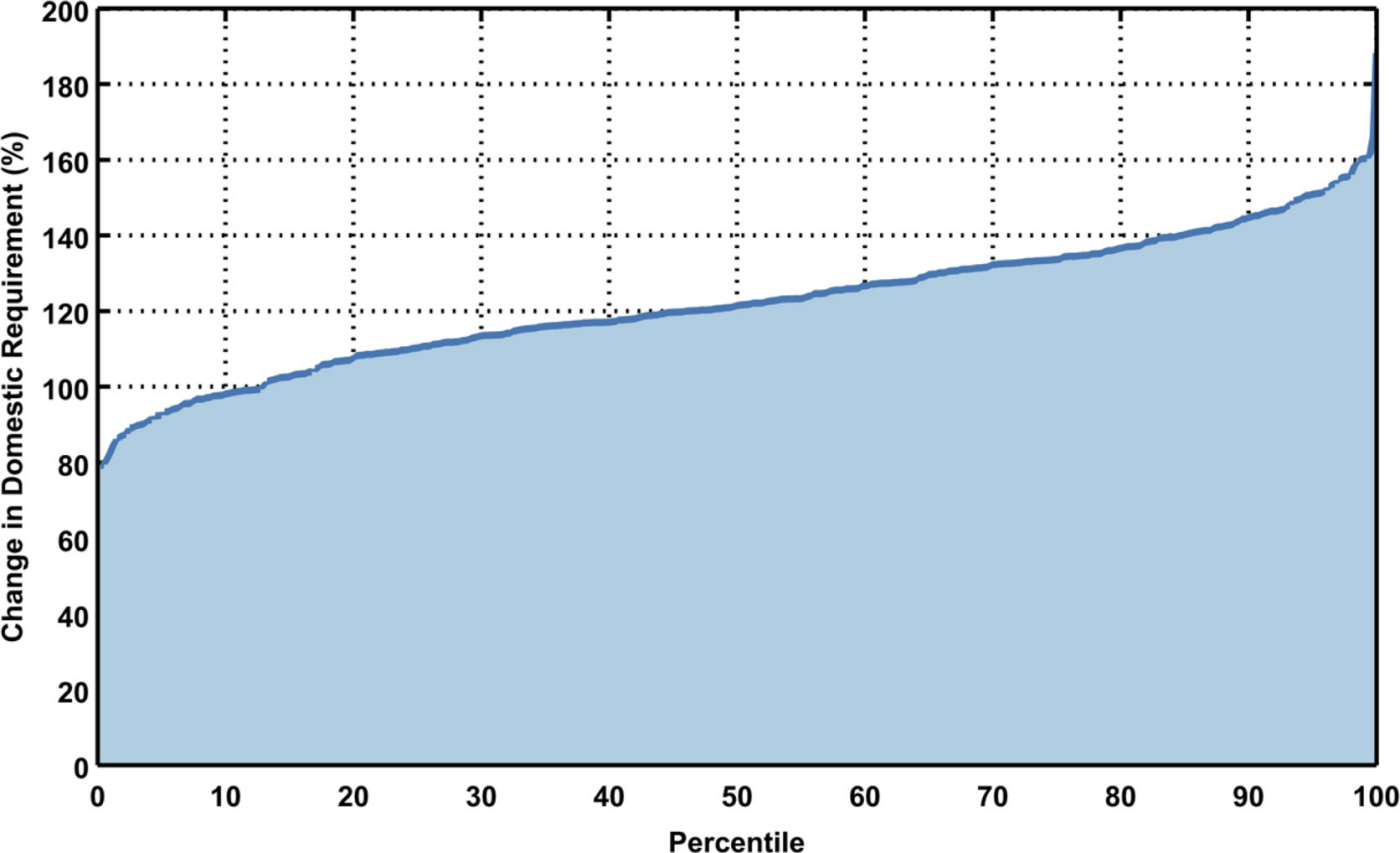
As in Fig 13, but for percent change in domestic requirement for the region for all scenarios. Each point in the line represents one of 400 growth scenarios. Percent change for each ASR in each scenario is weighted by population.

Domestic water requirement for the baseline scenario is shown in Fig 17. Taking a similar approach as with the runoff and irrigation requirements, a region-wide estimate of domestic water requirement is shown in Fig 18 as a percent change, weighting each ASR value by population. Compared to changes in irrigation requirement, domestic water requirement grows substantially percentage-wise, with an 80% to 180% increase. But, since the baseline domestic requirement is small compared to the baseline irrigation requirement (see Figs 11 and 16), the total amount of increase in requirement is relatively small.

**Fig 19:**
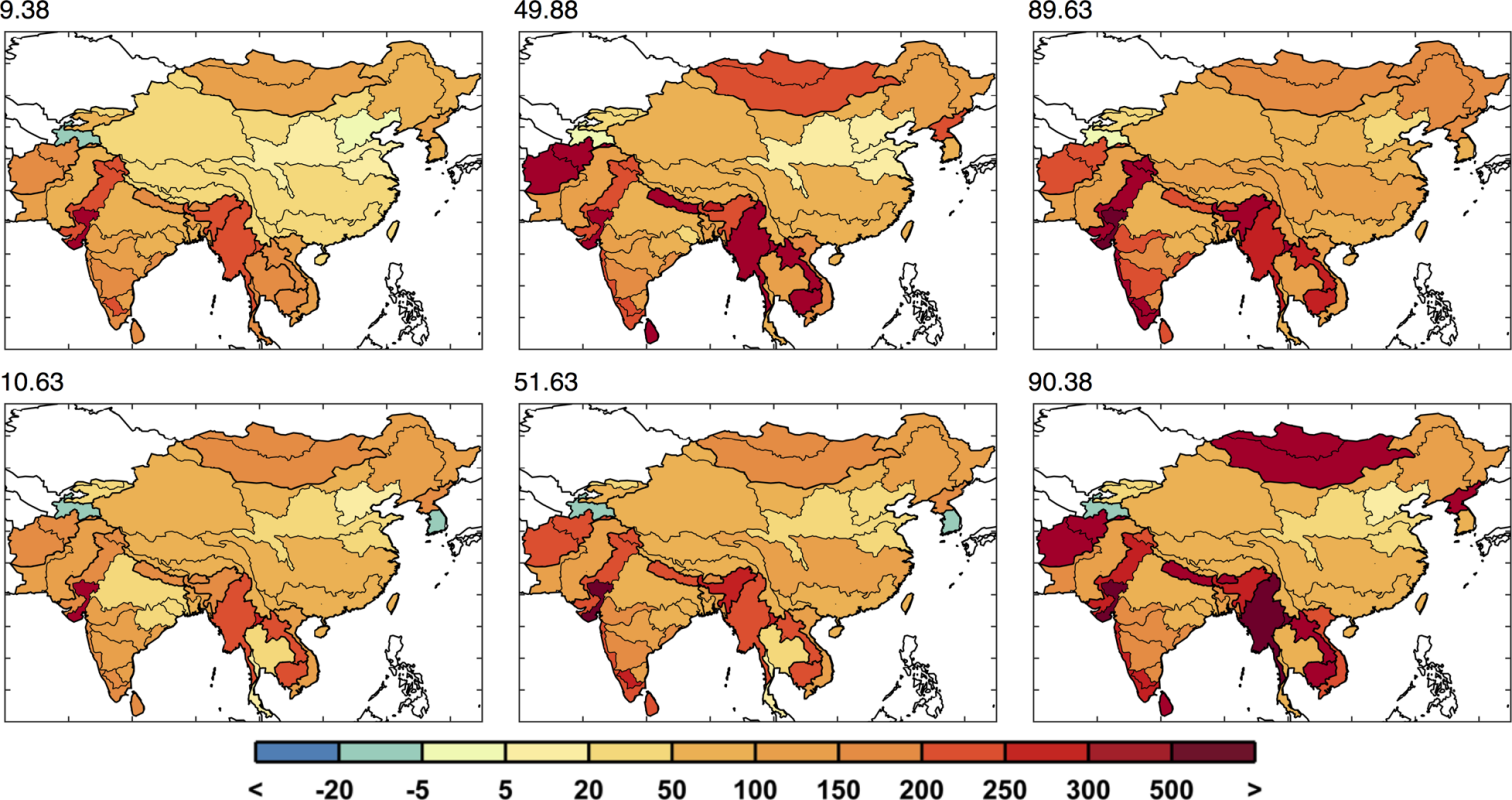
Domestic water requirement change by region (in %) around the 10^th^ percentile, median, and 90^th^ percentile, two each, similar to the metric used in Fig 18. Top label shows the percentile.

**Fig 20:**
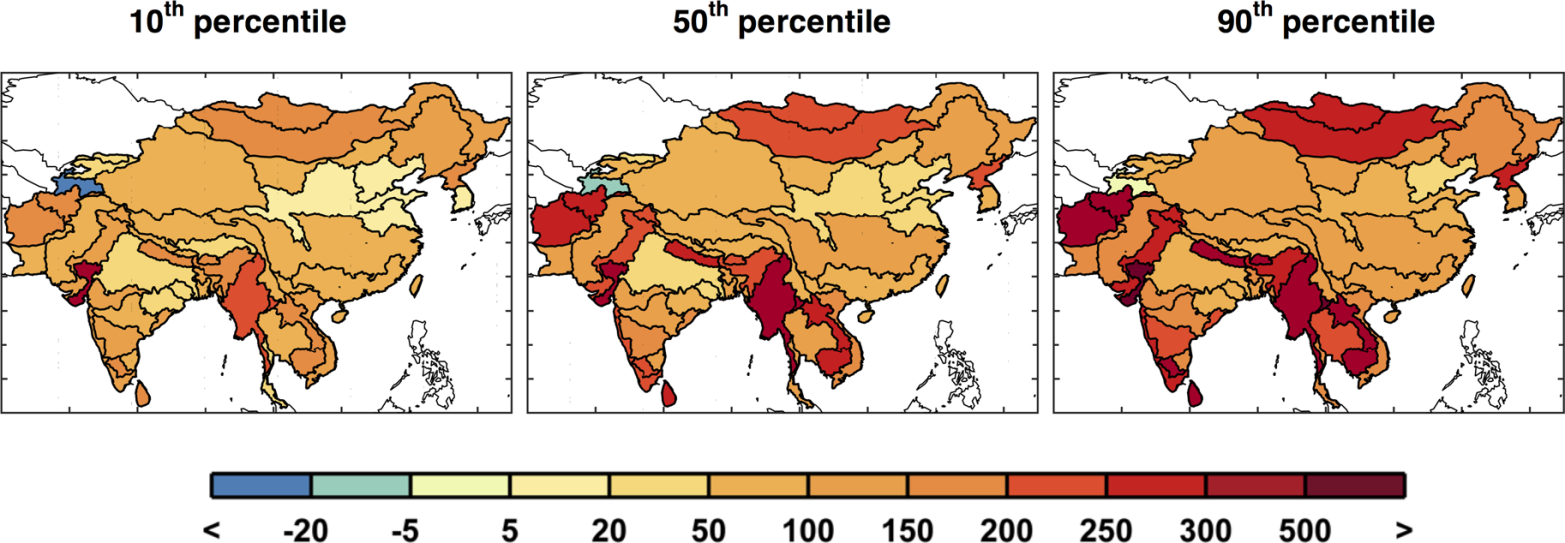
Changes from baseline in domestic water requirement (%) calculated point-wise by ASR, changes are based on the baseline (Fig 16) to the future scenarios averaged over 2041–2050 and shown for the 10^th^, 50^th^, and 90^th^ percentiles for each ASR.

Now we analyze the variety of domestic water requirement mapped across the region (shown in Fig 19). For the growth parameters, the variety of changes across the region is derived from the EPPA results and is important in order to be consistent in terms of the interaction of the region’s socio-economics by EPPA region (Fig 1). Fig 20 summarizes the distributional changes in domestic water requirement (2000–2050) across scenarios by presenting the 10^th^, 50^th^ and 90^th^ percentiles, calculated individually for every ASR. China is expected to see relatively small increases in domestic requirement compared to Mainland Southeast Asia and parts of India, where substantial increases are expected—between 2 and 5 times the baseline values.

#### 3.3.2 Industrial Water Requirement

**Fig 21:**
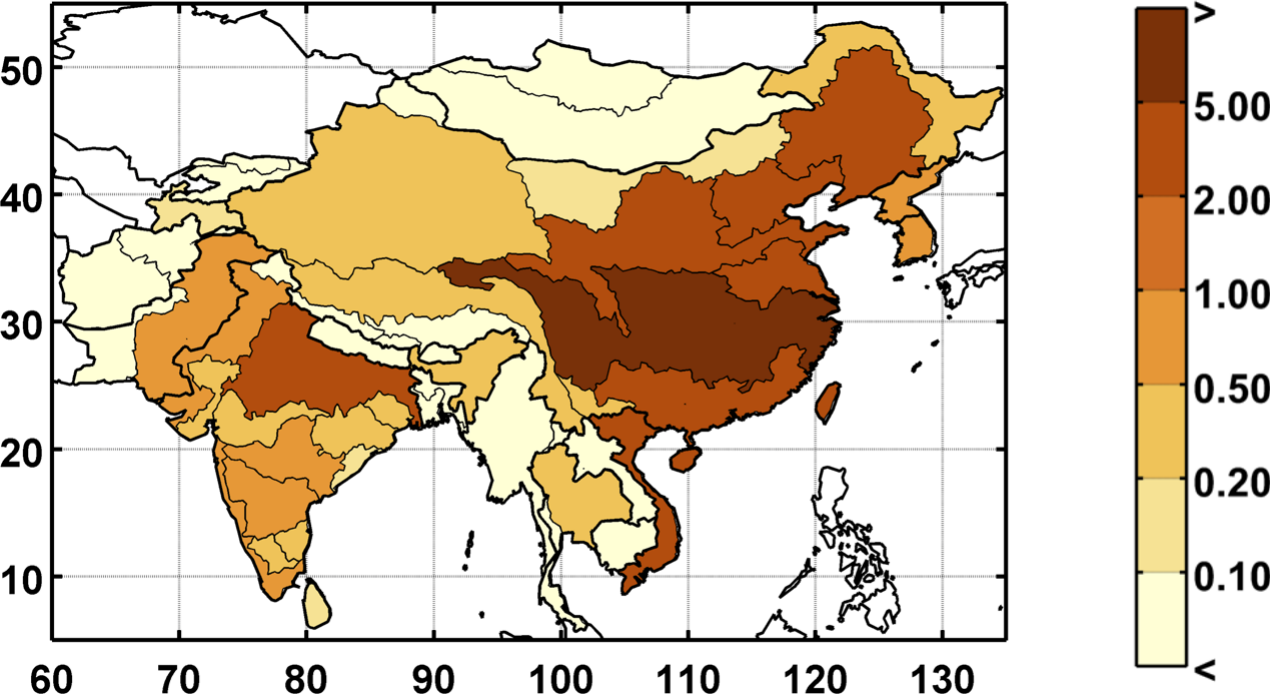
Baseline industrial water requirement (in billion cubic meters).

**Fig 22:**
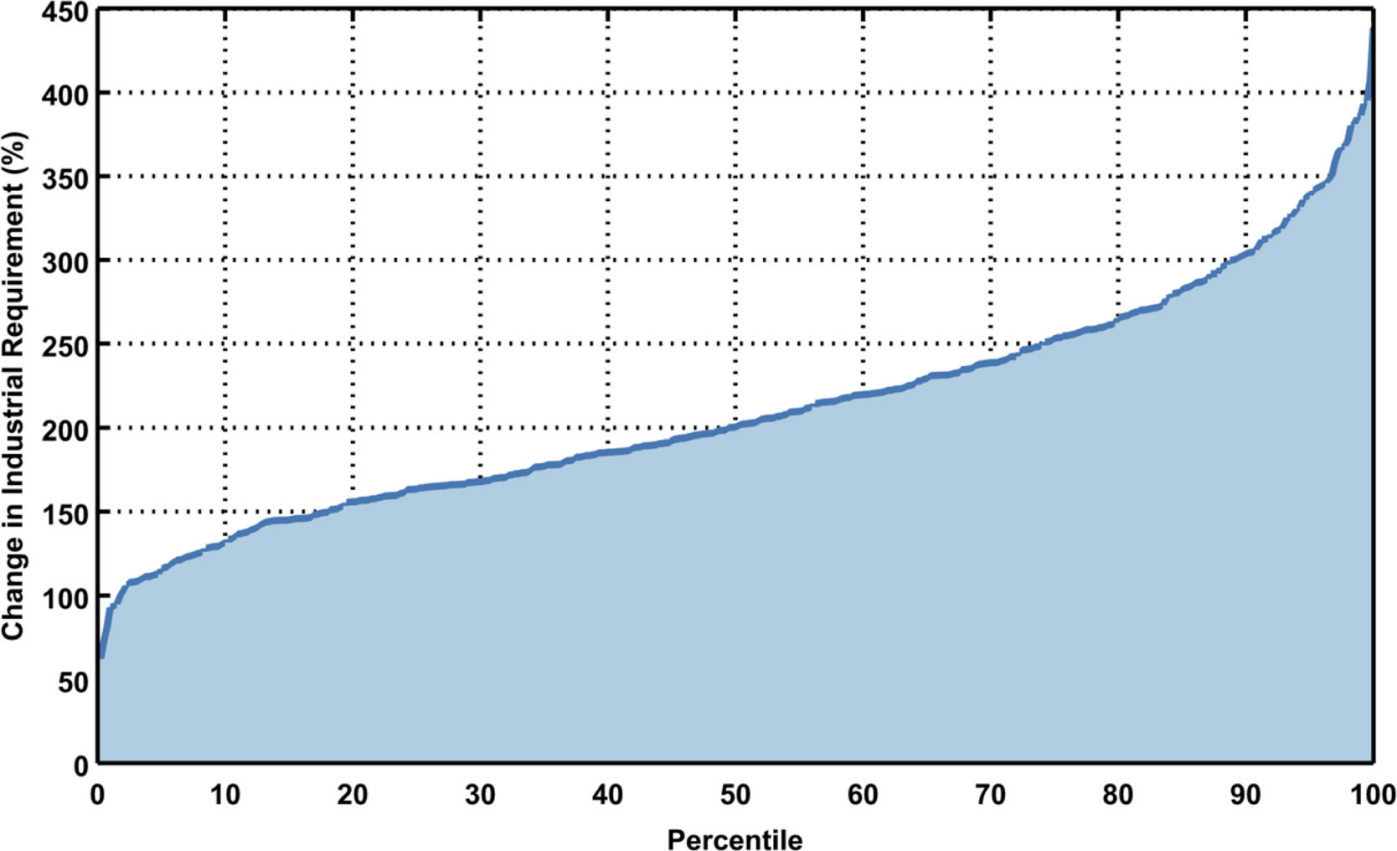
Mean change in industrial requirement for the region for all scenarios. Each point in the line represents one of 400 growth scenarios. Percent change for each ASR in each scenario is weighted by population.

**Fig 23:**
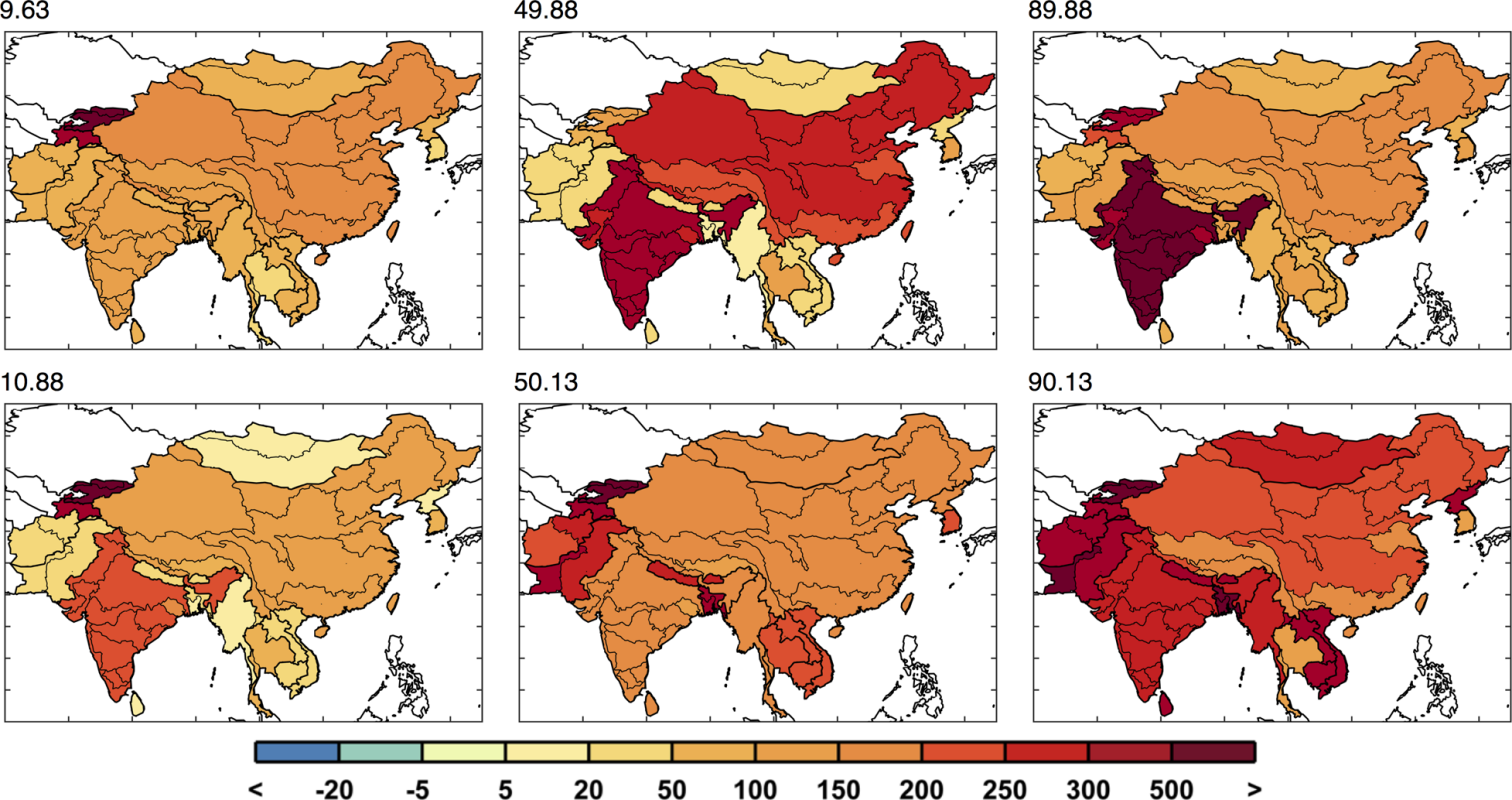
Industrial requirement change (in %) around the 10^th^ percentile, median, and 90^th^ percentile, two each, based on the mean industrial requirement change for the region (the metric used in Fig 22). Top label shows the percentile.

In this model framework, industrial water requirement, an aggregate of all industrial water use, responds to changes in per capita GDP. The baseline industrial water requirement is shown in Fig 21. A large portion of the industrial requirement is in China, with a fair amount in India and Vietnam. Fig 22 shows the inverse cumulative distribution of population-weighted percent change in industrial requirement. Industrial requirement varies considerably across scenarios, ranging from 60% increase to 440% increase from baseline, with a median of about 200%. In Fig 23, six examples of the variety of industrial water requirement changes are shown across scenarios of similar percentiles based on the mean percent change weighted by future population. Here we can see the richness of the scenario members’ patterns derived by the socio-economic modeling.

**Fig 24:**
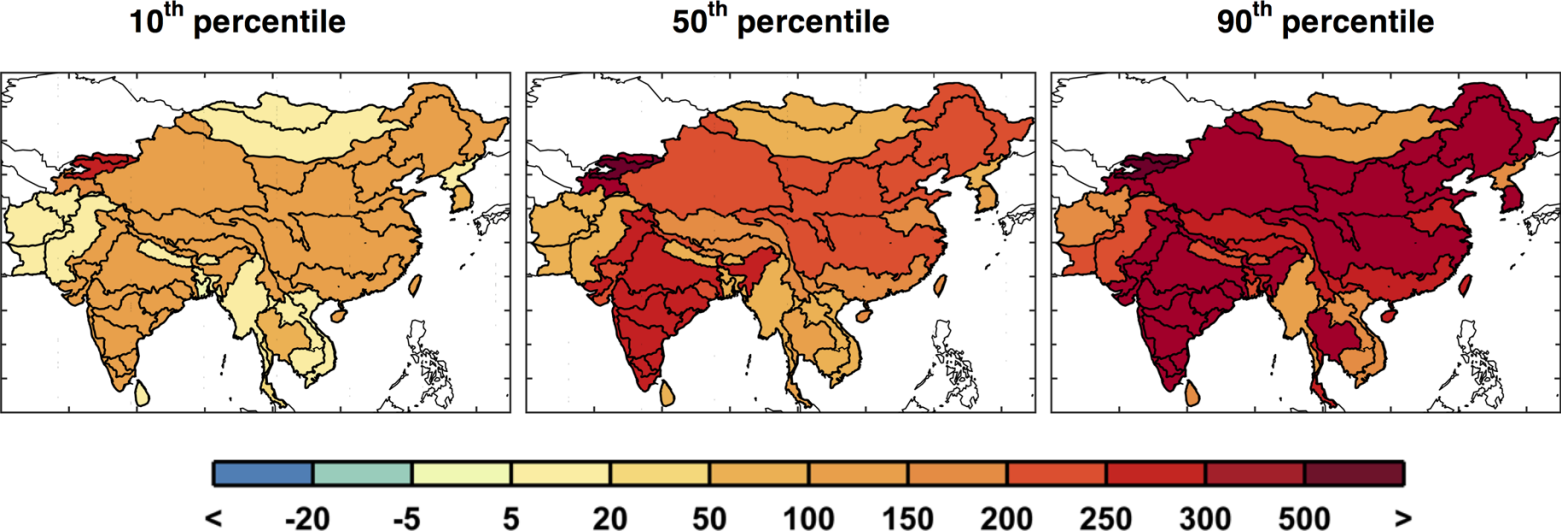
As in Fig 19, but for industrial water requirement shown for the 10^th^, median, and 90^th^ percentiles for each ASR.

Once again, using the probability distribution of each individual ASR, we calculate and map the 10^th^, median, and 90^th^ percentiles, shown in Fig 24. Here we see that the industrial requirement for ASRs in India and China increase considerably, by about two or three times the baseline amount in the median case and three to four times in the 90^th^ percentile.

### 3.4 Mapped Changes in Water Stress

As previously discussed (Section 2.4), we assess changes in water stress via two measures: UWR and WSI. We will also evaluate these changes across three ensembles: *Just Growth*, *Just Climate*, and *Climate and Growth*. For the *Just Growth* ensemble, we run the model with the baseline climate, changing only the growth parameters—population and GDP—which affect domestic and industrial water requirements. With this ensemble, we isolate the effects of growth by removing the effects of climate. In the *Just Climate* ensemble, we keep the growth parameters constant at the year-2000 value, and provide the model with a different future climate projection for each scenario. With this ensemble, we remove the effect of growth and focus on the effect of climate change. In reality, growth and climate occur simultaneously; however, for policy decisions, distinguishing growth effects from climate change effects is important since policy rarely targets both growth (e.g., population or wealth) and climate (GHG mitigation) simultaneously. We are also interested to assess the degree to which the effects of growth and climate interact non-linearly. Hence, we run a final ensemble, *Climate and Growth*, which combines the two effects, and represents the future we face under an “unconstrained emissions” pathway.

**Fig 25:**
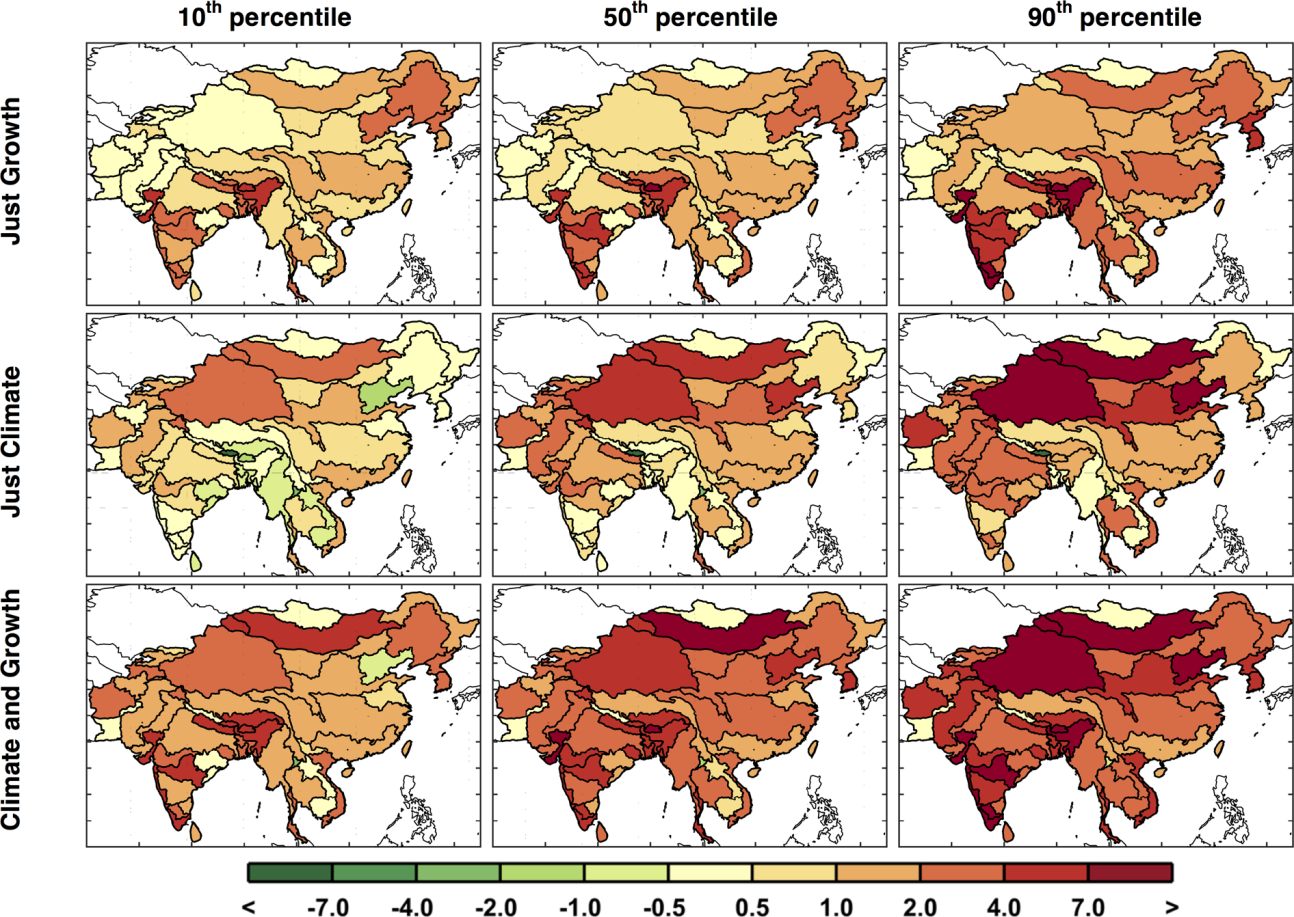
Exceedance changes in ASR UWR (%). Changes are based on the baseline (Fig 6) to the future scenarios averaged over 2041–2050 and shown for the 10^th^, 50^th^, and 90^th^ percentiles for each ASR.

In Fig 25 the 10^th^, 50^th^, and 90^th^ percentiles of UWR point-wise distribution are mapped for each of the three ensembles. As shown, UWR rarely decreases in the future. For the *Just Growth* ensemble, we see UWR increasing or remaining constant, even in the 10^th^ percentile, although there are some basins that are affected by growth changes more than others, especially much of India where increases are the most substantial. In the *Just Climate* ensemble, driven by the changes in runoff and irrigation requirement, UWR increases for much of China (especially in the north), Afghanistan and Pakistan, as well as northern India. For the *Climate and Growth* median case, we find a fairly consistent increase across the region with only a few ASRs showing no change. Essentially, this third ensemble appears to be a simple sum of the two above, where, for example, in India, growth has larger effects in the south and climate in the north resulting in a relatively evenly distributed stress across the country. UWR, which is based on the ratio of consumption over demand, will behave this way (i.e. as a simple summation) when the modeled basin management is not able to increase consumption even though demand increases. This result implies that adaption or mitigation to water shortage will be a necessity in the region. Appendix A shows the distribution of scenarios using the population-weighted change in UWR and the example maps around specific points in the distribution using that single metric.

**Fig 26:**
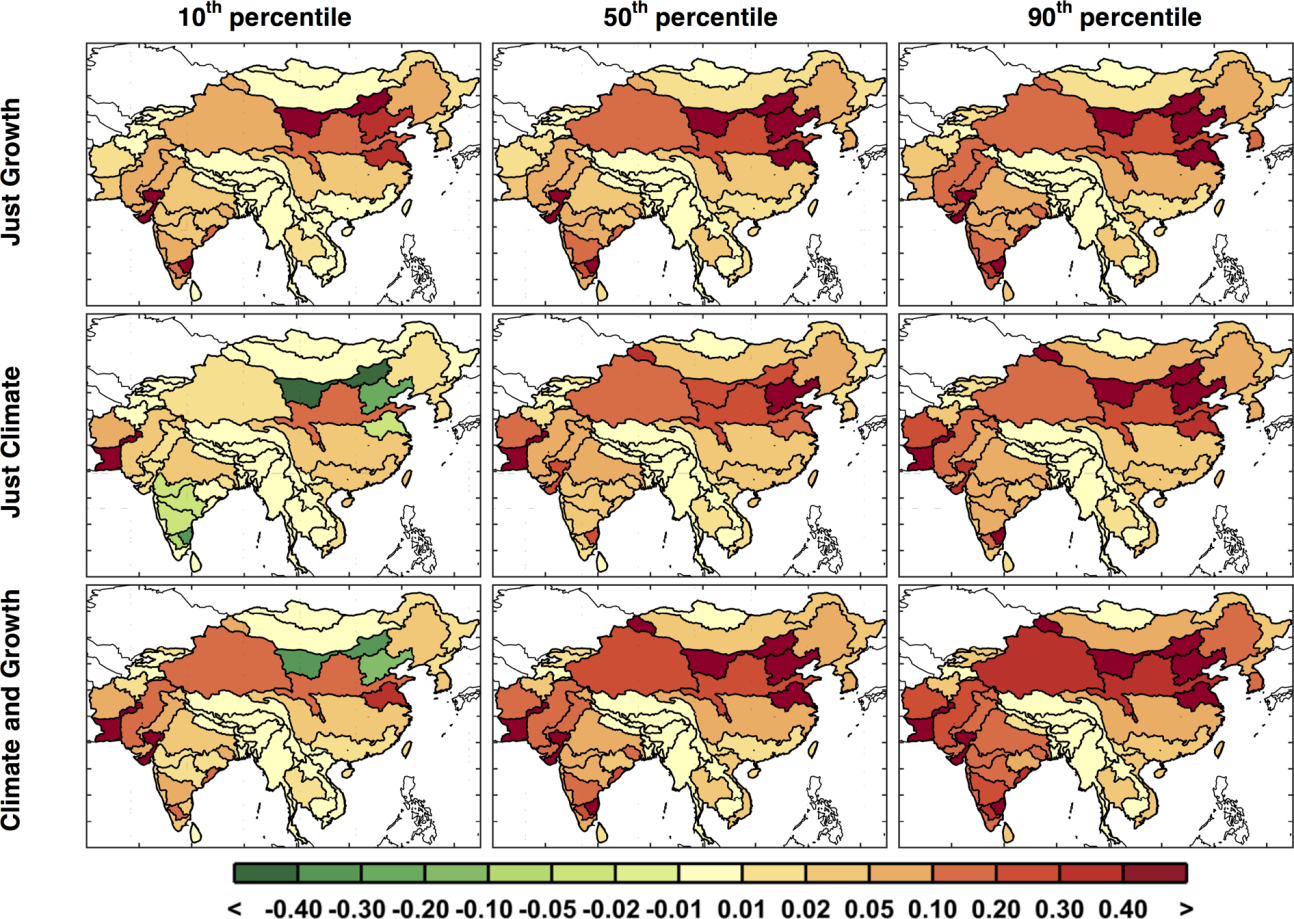
Exceedance changes in decadal averaged WSI (unitless). Changes are based on the baseline (Fig 7) to the future scenarios averaged over 2041–2050 and shown for the 10^th^, 50^th^, and 90^th^ percentiles for each ASR.

Fig 26 shows series of maps for the WSI similar to that shown for UWR in Fig 24. Results for the three ensembles are mapped for every ASR based on specified exceedances—10^th^, 50^th^, and 90^th^ percentiles. In the *Just Growth* ensemble we find that northern China and southern India are most prone to increased stress caused by growth. And we see a similar pattern in the *Just Climate* ensemble, although for India the climate effect is fairly evenly distributed. Increases in stress are also prominent in the west, in Pakistan and Afghanistan. We see these same patterns emerging, although more pronounced, in the final ensemble, *Climate and Growth*. WSI, as apposed to UWR, does not change directly by changes in demand, but responds to changes consumption and changes in water supply. So, if consumption cannot increase by better reservoir management, WSI will respond only to changes in runoff. By comparing these results to the changes in runoff shown in Fig 12, we can see that for northern China, as well as for Pakistan and Afghanistan, where runoff decreases are more substantial, this appears to be the case. For much of India, however, these changes in WSI appear to be driven by an increase in consumption rather than changes in runoff, and therefore the water resources are further exploited by more efficient basin management.

### 3.5 Water Stress Frequency Distributions

Next, we show the frequency distributions of outcomes to qualify the consequences of the scenarios in terms of future risk. We select specific regions, first by political boundaries and second by hydrologic basin boundaries, and calculate the aggregate water stress for 2041–2050 over the region, with ASR values weighted by population. As in the previous section, we compare these WSI changes with the baseline scenario WSI. Recognizing that the baseline scenario is only one of many possible traces of climate through time, we develop a *baseline ensemble* to understand the range of water stress that results from the impact of climate sequences on water availability. To do this, we use a multivariate k-nearest-neighbor bootstrap approach (as discussed in [39]) to develop this baseline ensemble of 500 members, each containing a 50-year time series of the required WSM climate-dependent input data including monthly values of irrigation requirement, runoff, and reservoir evaporation. [39] shows that the multivariate k-nearest-neighbor bootstrap approach has the advantage over simple bootstrapping in that it maintains the lag-1 correlation as well as geospatial correlations. As constructed, the baseline ensemble can be viewed as a statistically based emulation of the uncertainty in the WRS projection caused by the natural variability of climate–which is inherently unpredictable.

**Fig 27:**
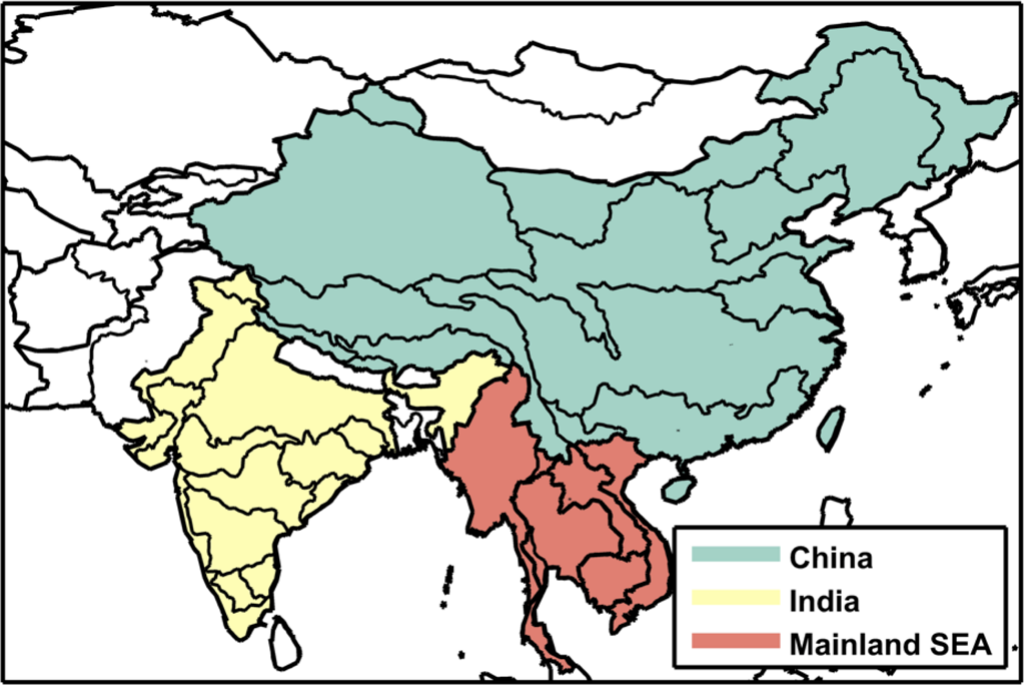
Map of major political regions showing the aggregate frequency distributions of water stress.

**Fig 28:**
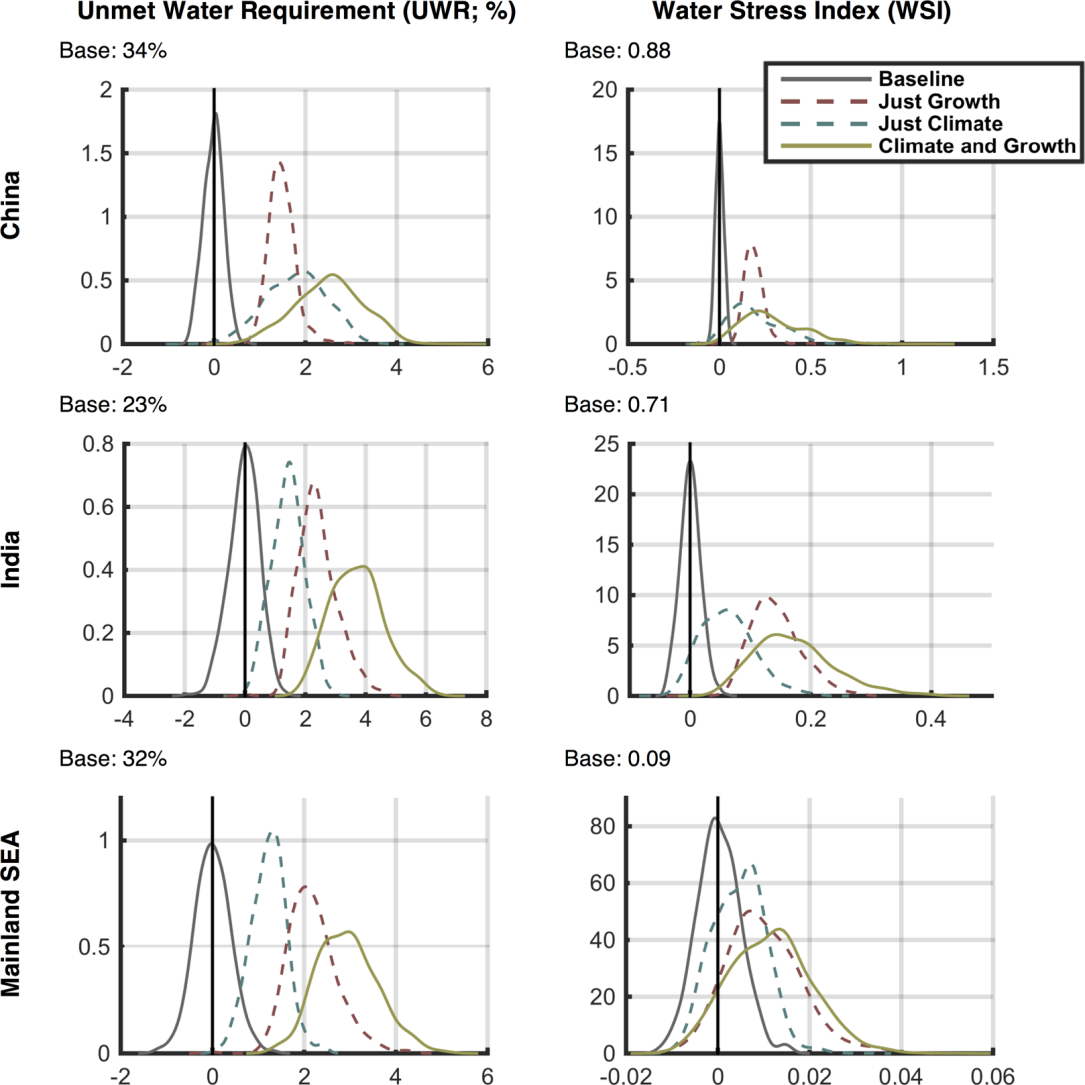
Frequency distributions of changes in decadal averaged Unmet Water Requirement (UWR, left column) and water stress index (WSI, right column) for 2041–2050 against the baseline result aggregated over major socio-economic regions (Fig 28) and weighted by population. Mean baseline value shown above each figure. Results are shown for the Just Growth, Just Climate, and Climate and Growth ensemble scenarios. In addition, a distribution for the Baseline result is also provided that depicts the range of UWR and WSI decadal-averaged changes that would result from internal variability of the climate forcing (see text for details).

The distributions of the two stress indices for China, India, and Mainland Southeast Asia (as shown in Fig 27) are shown in Fig 28, where a kernel smoothing approach is used to approximate the shape of the distributions. These plots show characteristics of the distributions, e.g., mode, skewness and the nature of the distribution tails, all of which illustrate the likelihoods associated with the respective impacts. The three future ensembles—*Just Growth* in red, *Just Climate* in blue, and *Climate and Growth* in yellow—are shown as the difference from the last decade of the baseline scenario value (2040–2050) and that of the future result. The baseline ensemble distribution (in grey) shows the difference between the 50-year baseline scenario mean and the last ten years of each baseline ensemble member. The baseline scenario-mean value is also printed above each plot. We can thus compare the distributions from natural variability (the grey distribution) to the range of the future water stress ensembles to understand the magnitude of the uncertainty derived from changes in climate, growth, or both. Note that we remove the natural variability from the changes in these future WSIs by comparing them with the baseline, which contains the same natural variability. This is an effect of using the delta method, and allows us to focus on long-term mean changes, isolating the effect of the climate change trend from that of natural variability in climate. In China, for UWR, both growth and climate have adverse effects but climate is slightly stronger with a noticeable widening of the distribution, i.e. more climate uncertainty than growth. The *Climate and Growth* ensemble is, as expected, worse but not simply an aggregate of the two. The noticeable differences for WSI are that the mode of growth is more potent that climate; however, climate shows a noticeable tail on the side of high impact. In India, climate is less stress inducing than growth. In fact, for WSI, there are a few scenarios projecting positive impacts. Growth has a more adverse effect. We also see the long tail toward higher stress in the WSI plot although the maximum change in stress is not as high as for China. In Mainland Southeast Asia, both stress indices show a similar pattern to the distributions shown for India—likely from being in similar latitudes. Note that the natural variability distributions are rarely wider than the distribution of the future ensembles, but in some cases (e.g., WSI in Mainland Southeast Asia) future uncertainty by the 2040s is close to the historical uncertainty.

**Fig 29:**
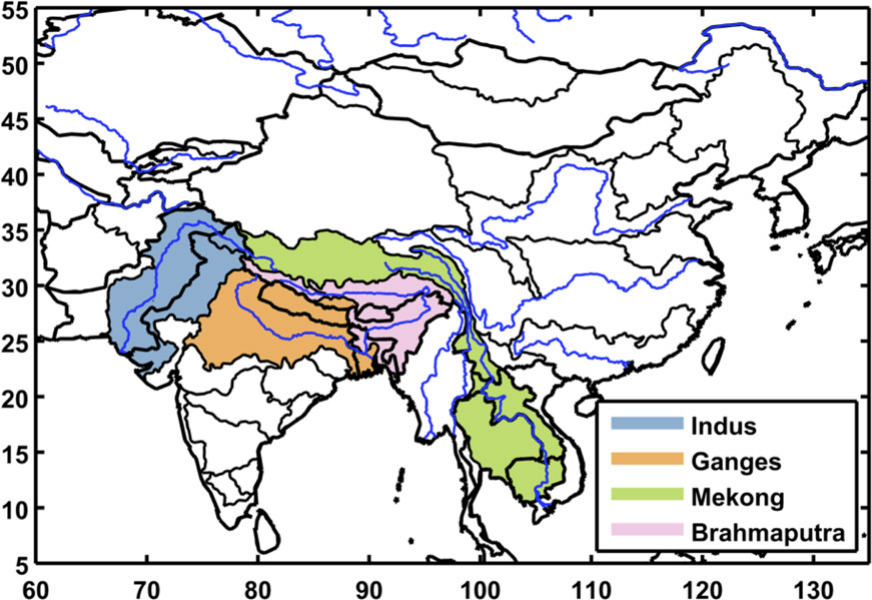
Map of major basins used to show the aggregate frequency distributions of water stress.

**Fig 30:**
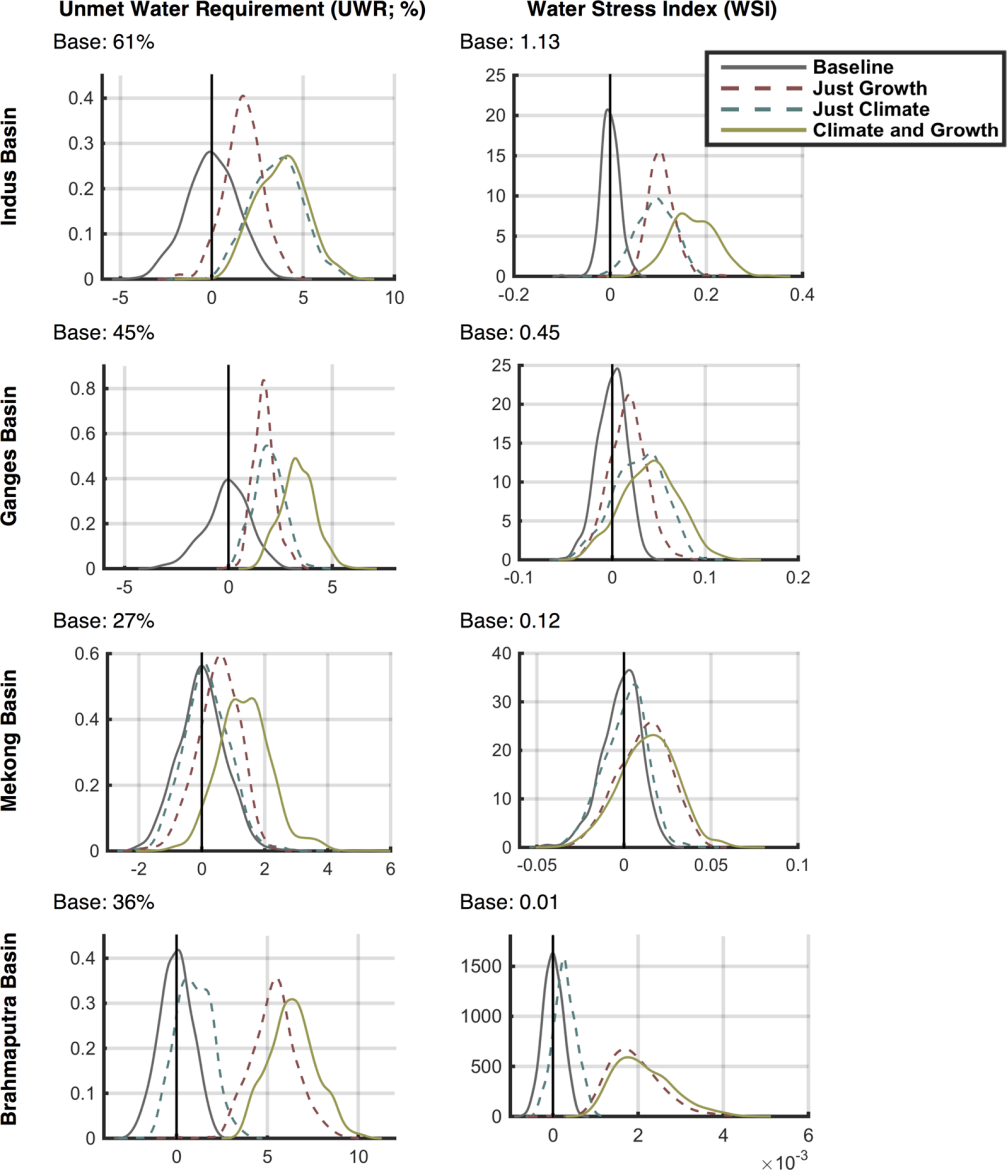
Frequency distributions of changes in decadal averaged Unmet Water Requirement (UWR, left column) and water stress index (WSI, right column) for 2041–2050 against the baseline result aggregated over selected basins (Fig 30) and weighted by population. Mean baseline value shown above each figure. Results are shown for the Just Growth, Just Climate, and Climate and Growth ensemble scenarios. In addition, a distribution for the Baseline result is also provided that depicts the range of UWR and WSI decadal-averaged changes that would result from internal variability of the climate forcing (see text for details).

Next, we aggregate by hydrologic basin (see Fig 29), again using population to give each ASR a respective weight. These basins were chosen because their rivers cross country boundaries and could be a cause for political tension. As seen in Fig 30, in the Indus Basin, shared mostly by India and Pakistan, there is considerable stress in the baseline case; climate and growth both increase stress further, and few scenarios result in a stress decrease. In the Ganges basin, climate and growth both increase stress for most of the scenarios; growth and climate are both about equally potent. The Mekong and Brahmaputra basins both reside in wet climates with low storage basin-wide. These areas are also major producers of paddy rice, a water intensive crop, which results in high UWR. In the Mekong, climate has about an equal chance of either increasing or decreasing stress, and the growth effects are minor; in the Brahmaputra, climate has a slightly negative, almost neutral effect, while growth is more extreme—especially in terms of UWR changes.

### 3.6 Populations at Risk to Increased Water Stress

**Fig 31:**
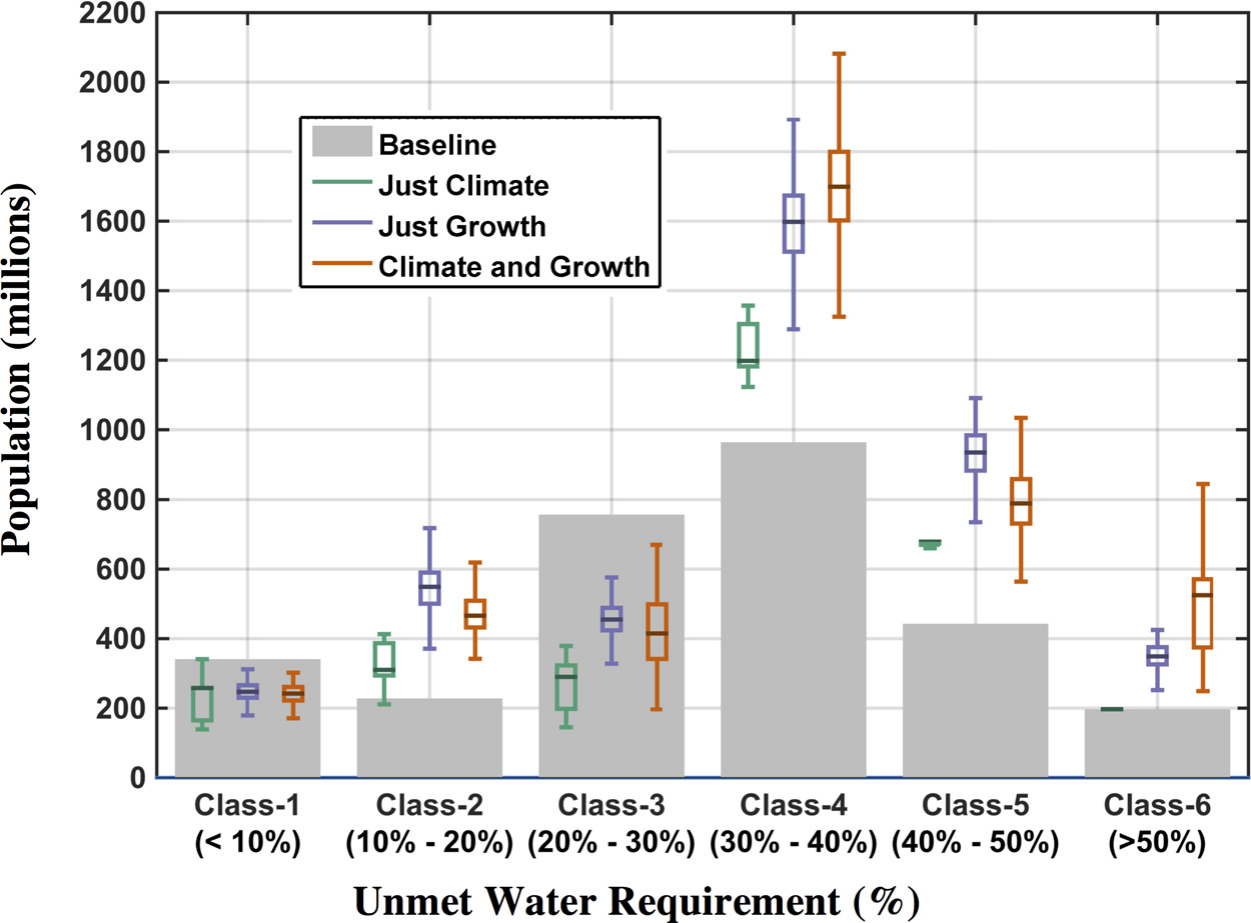
Population exposed to water stress based on UWR classifications using the 2041–2050 mean. Grey bars represent the number of people in each class in the baseline scenario (set to year-2000 value); the box-and-whisker plots show the distributional characteristics of the three ensemble scenarios.

An analysis was performed to assess the population that is prone to water-stress exposure under current conditions and future scenarios. The population of each ASR was assigned to one of the water-stress classifications, using both UWR and WSI, based on the value of the resulting water stress indicator. Note that in our model we are assuming that population growth is constant by EPPA region, where each ASR grows proportionally to the baseline population. For UWR, a simple classification is used: Class 1 is less than 10%, class 2 is between 10% and 20%, classes 3, 4 and 5 are also set at increments of ten, and the final class, 6, is set to values above 50%. We count the number of people in each UWR class for the Baseline scenario and compare that to the same metric at 2050 from the three scenario ensembles (*Just Climate*, *Just Growth*, and *Climate and Growth*). These results are shown in Fig 31.

In this figure, note that the *Just Climate* ensemble has no population growth, so the difference between the *Just Climate* and the other two ensembles is largely attributed to the additional population (in the *Just Growth* and *Climate and Growth* scenarios). Also, note that the *Just Climate* ensemble does not change much in the extreme classes: Class 1 and 6. The largest effect of climate is to decrease population in Class 3 and increase in Class 4 (see Table 1), which is a movement into a more severe water stress state. We see this movement in *Just Growth* as well (Table 1), but here the unmet requirement is changing for many ASRs from Classes 2 and 3 into the next higher stress class (Classes 3 and 4, respectively). These ASRs are moving into a higher UWR class because *growth only* increases UWR, as we have shown (Section 3.5). The most striking change is the increase of population in Class 4 and 5, compared to the baseline, for both the *Just Growth* and *Climate and Growth* ensembles. The increases in Class 6 are largely attributed to population growth occurring within ASRs that are already at this high stress level (Table 1); increases in Class 4 populations are largely attributed to the addition of former Class 3 populations, due to water demand increases from growth (Table 1) or the combined effects of growth and climate change on supply and demand changes (Table 1). An additional notable result is that there are very few instances of populations moving to decreased stressed conditions, seen in a small fraction of cases for the *Just Climate* scenario in the 90^th^ percentile (Table 1).

**Table 1:** Matrix of populations’ (in millions) exposure to water stress. Shaded gray cells show the population remaining in the UWR class relative to the Baseline result. The off-diagonal cells denote population shifts by 2050 across the various UWR classes; population shifts between classes are depicted by their location within the table matrix. Each cell provides the 10^th^ [left, bracketed], 50^th^ (center), and 90^th^ [right, bracketed] percentile results.

**Just Climate**						
From \ To	Class-1	Class-2	Class-3	Class-4	Class-5	Class-6
Class-1	[165] **259** [341]	[1] 84 [177]				
Class-2		[212] **212** [212]	[17] 17 [17]			
Class-3		[0] 0 [16]	[165] **288** [307]	[451] 454 [576]		
Class-4				[727] **727** [744]	[219] 236 [236]	
Class-5				[0] 0 [2]	[440] **442** [442]	
Class-6						[198] **198** [198]
**Just Growth**						
From \ To	Class-1	Class-2	Class-3	Class-4	Class-5	Class-6
Class-1	[214] **247** [280]	[213] 241 [269]				
Class-2		[211] **311** [367]	[0] 49 [153]			
Class-3			[362] **409** [448]	[585] 689 [801]		
Class-4				[800] **898** [1003]	[237] 272 [308]	
Class-5					[550] **664** [726]	[0] 0 [178]
Class-6						[306] **347** [392]
**Climate and Growth**							
From \ To	Class-1	Class-2	Class-3	Class-4	Class-5	Class-6
Class-1	[204] **242** [277]	[215] 246 [286]				
Class-2		[172] **209** [313]	[50] 160 [193]			
Class-3			[161] **274** [422]	[648] 808 [976]		
Class-4				[788] **890** [998]	[239] 272 [310]	
Class-5					[426] **505** [632]	[34 185 [259]
Class-6						[305] **348** [393]

**Fig 32:**
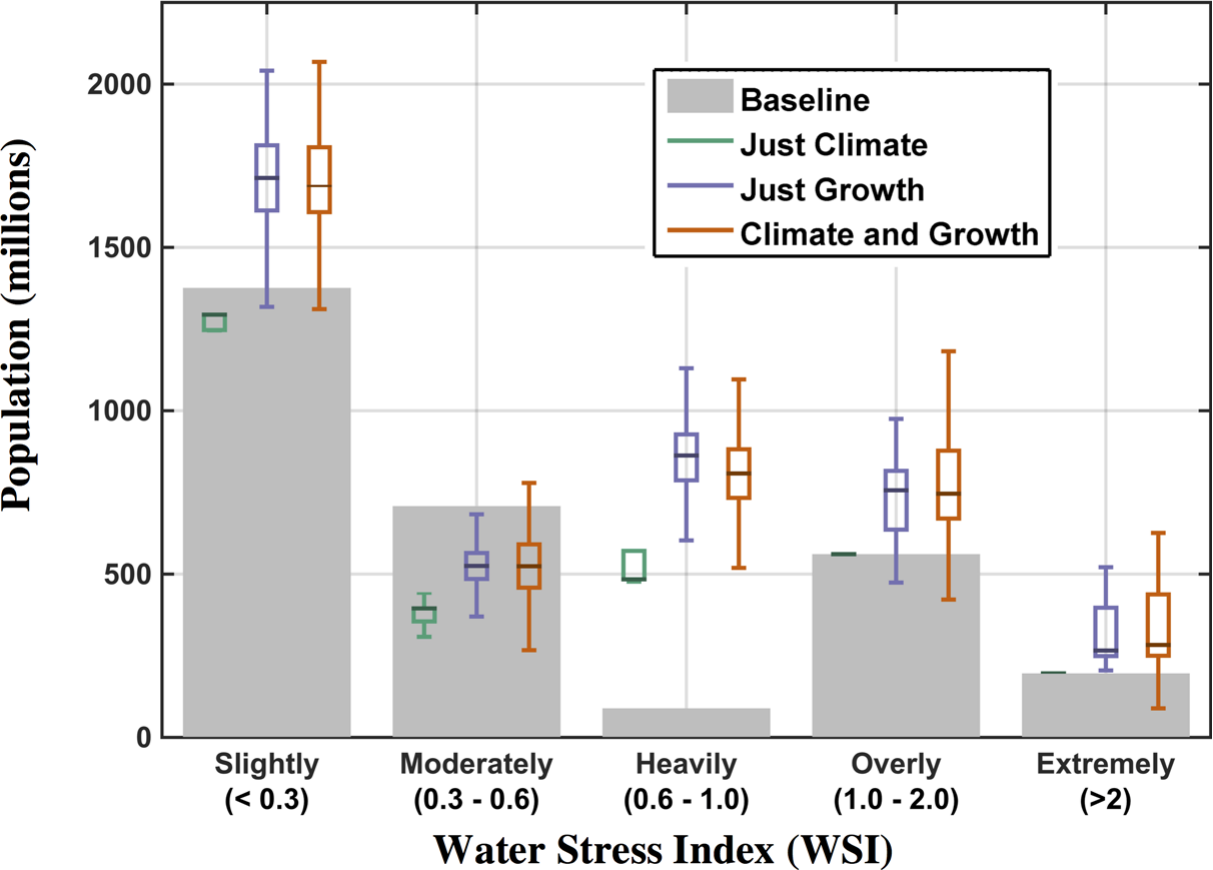
Population exposed to water stress based on WSI classifications using the 2041–2050 mean. Grey bars represent the number of people in each class in the baseline scenario (set to year-2000 value); the box-and-whisker plots show the distributional characteristics of the three ensemble scenarios.

For WSI, water-stress classifications are based on the aforementioned [38] study (Section 2.4): *WSI* < 0.3 is slightly exploited; 0.3 ≤ *WSI* ≤ 0.6 is moderately exploited; 0.6 ≤ *WSI* ≤ 1 is heavily exploited; 1 *≤ WSI* < 2 is overly exploited; and *WSI* ≥ 2 is extremely exploited. The strongest effect of the *Just Climate* scenario is to bring more populations currently living under Moderately stressed conditions into Heavily water stressed environments by 2050 (Fig 32, Table 2). Similar to the results seen for UWR, there are only a small number of cases, shown in the 90^th^ percentile, in which climate will move populations into less stressed WSI conditions. The effect of *Just Growth* is consonant with the *Just Climate* result (Table 2), where a comparable increase in population is taken from Moderately into Heavily stressed conditions. Combined, the *Climate and Growth* scenario places comparable populations from Moderately stressed environments into Heavily stressed environments (Fig 32, Table 2). In doing so, the Moderately stressed condition is the only class of WSI that contains a decrease in the median total population (on the order of 200 million) at 2050 (compared to the Baseline condition).

**Table 2:** Matrix of populations’ (in millions) exposure to water stress. Shaded gray cells show the population remaining in the WSI class relative to the Baseline result. The off-diagonal cells denote population shifts by 2050 across the various WSI classes; population shifts between classes are depicted by their location within the table matrix. Each cell provides the 10^th^ [left, bracketed], 50^th^ (center), and 90^th^ [right, bracketed] percentile results.

**Just Climate**					
From \ To	Slightly	Moderately	Heavily	Overly	Extremely
Slightly	[1249] **1296** [1378]	[0] 82 [129]			
Moderately		[226] **313** [313]	[395] 395 [482]		
Heavily			[89] **89** [89]		
Overly			[0] 0 [26]	[535] **562** [562]	
Extremely				[0] 0 [122]	[74] **196** [196]
**Just Growth**					
From \ To	Slightly	Moderately	Heavily	Overly	Extremely
Slightly	[1531] **1713** [1875]	[136] 177 [318]			
Moderately		[245] **329** [393]	[637] 754 [872]		
Heavily			[14] **114** [133]	[0] 25 [108]	
Overly				[538] **736** [813]	[0] 0 [194]
Extremely					[228] **254** [278]
**Climate and Growth**					
From \ To	Slightly	Moderately	Heavily	Overly	Extremely
Slightly	[1534] **1693** [1882]	[110] 180 [324]			
Moderately		[234] **313** [404]	[624] 767 [901]		
Heavily			[11] **14** [125]	[20] 117 [143]	
Overly				[526] **657** [801]	[0] 1 [209]
Extremely				[0] 0 [132]	[117] **249** [276]

We further aggregate these classifications to underscore the impacts of these scenarios on the more severe water-stress conditions. We assign a threshold to both the UWR and WSI measures, so an ASR may be classified as either stressed (over the threshold) or unstressed (under the threshold). The developed aggregations are shown in Table 3. For UWR we use a threshold value of 30% (reflecting at least 30% annual water requirements not met) and for WSI we use a threshold value of 0.6 (must be at least in the Heavily stressed class). Overall, we find no occurrences (in any member, among all scenarios) of a decrease in the total water-stressed population by 2050. The effect of socio-economic growth is quite evident, as seen by the median result of over 1 billion additional people exposed to “water-stressed” conditions by 2050. Additionally, in only 10% of the members will this result be below 850 million based on either the UWR or WSI indicators. The *Just Climate* scenario impact is smaller than the others, causing a median shift of about 13–16% population increase. The *Just Growth* scenario also provides very comparable results between the UWR and WSI based thresholds—both outcomes indicating that over 1 billion additional people by 2050 will become water-stressed due to socioeconomic growth unconstrained by global actions to limit greenhouse gas concentrations and autonomous adaptation. There is a small adverse effect to this result when the effects of climate change are added (as indicated by the *Climate and Growth* result), but it only results in a ~1–3% increase in the population growth affected by socioeconomic changes. Overall, the central tendency of the UWR and WSI based thresholds in the *Climate and Growth* scenario is to flank (i.e. within ± 200 million) a future outcome that 1 billion additional people will be living in regions under water stress.

**Table 3:** Water-stressed population increase (in millions and percent). Based on a threshold of 10% for unmet requirement (UWR) and 0.6 for WSI. Each cell provides the 10^th^ percentile [left bracketed value], median (center in bold type), and 90^th^ percentile [right bracketed value] results.

	UWR > 30% [Baseline = 1604 / 55%]	WSI > 0.6 [Baseline = 846 / 29%]
	[10^th^] Median [90^th^]	[10^th^] Median [90^th^]
*Just Climate*	[451] **454** [576] / [15.4%] **15.5%** [19.6%]	[395] **395** [482] / [13.5%] **13.5%** [16.4%]
*Just Growth*	[1027] **1301** [1507] / [27.0%] **31.0%** [34.0%]	[866] **1055** [1231] / [22.7%] **25.5%** [27.9%]
*Climate and Growth*	[1105] **1393** [1694] / [29.1%] **33.5%** [38.4%]	[843] **1066** [1251] / [22.3%] **25.7%** [28.6%]

## 4. Discussion and Closing Remarks

This study has employed the IGSM-WRS framework aimed at assessing the fate of managed water systems, depicted by 52 large sub-regions across Asia. A number of experiments were performed to assess the isolated as well as combined effects of socioeconomic growth and regional climate change out through the 2040s. With this large ensemble of projections, a frequency distribution of impacts was developed that articulates the severity and likelihood of water stress in this region of Asia. We find a variety of patterns across the region for changes in surface-freshwater supply, and this results from a variety of influences in the hydroclimate (i.e. runoff) as well as water requirements in agriculture, industry and municipality water needs. We find that regions most vulnerable to changes in climate include much of China (especially in the north), Pakistan and Afghanistan. Further, India, China and Mainland Southeast Asia are all highly likely to experience significant changes in socioeconomically-driven water requirements.

Some limitations and assumptions made within the framework for this particular experimentation are notable. First, we model *consumptive* water requirement rather than withdrawal. We do this because our sub-regions are large, and presumably will include substantial water reuse within an ASR. Second, we keep irrigated areas constant by crop for all scenarios. In reality, these changes respond to a variety of drivers, including local and global food prices, land and water availability, and government subsidies. Estimating changes in irrigated area is a difficult task; however, we do plan to include these changes in an upcoming report, as they have the potential for profound impact on future water stress. Third, the framework uses socioeconomic drivers exogenously, therefore missing potentially important feedbacks (e.g., water limitations could have an adverse effect on food or energy prices, which would likely cause shocks in the economic system on a local or global scale, further affecting investments in water-related technology and infrastructure). The fourth limitation has to do with the treatment of uncertainties. As discussed in the introduction, we do not address uncertainty outside of emissions driven by economics, climate, population, and GDP growth. Lastly, this framework’s water management simulation uses a single objective function with perfect foresight within a calendar year, consistent priorities across all ASRs, and perfect cooperation within a river network. Furthermore, we do not simulate hydropower generation in the representative reservoirs. Realistic water management is done in a complicated, often inefficient fashion with varying knowledge about next month’s water supply (e.g., water managers generally know historical averages). These assumptions about water management allow us to have a water allocation scheme that is consistent across ASR and scenario, providing a model environment that is not partial to regions that are historically “better” water managers. Our scheme also adapts to changes in water supply and requirement more efficiently than a realistic system, likely providing a more optimistic picture of water stress in the region. For example, water managers are typically challenged and forced to make operational decisions based on imperfect information and forecasts, and hydropower needs may interfere with water allocation. These assumptions and limitations are issues we plan to address in future studies.

Regardless, there are a number of significant results we have found in this study. For example, by isolating socioeconomic growth from changes in climate, we find that the two have characteristically different impacts on water stress. Industrial and municipal water requirements, driven by socioeconomic growth, are less significant in the baseline but *will* increase considerably in the future. Alternatively, changes in climate can be significant in our current system, but do not change as much (compared to growth) in the future relative to their baseline value. Socioeconomic drivers on water requirements are, therefore, likely to play a larger role in future management decisions than they do in the current system. When we assess potential population increase in water stressed regions, we find it highly probable that many people who live in moderately stressed conditions will live in heavily stressed conditions in the future. We specifically find that increases in water-stressed populations will be about 1 billion in the median case, more than doubling the baseline case (for WSI). These changes will likely require more aggressive water policies and regulations in areas where water resource decisions have been less tense historically. Without assertive water policies in these regions, water limitations could be harmful to the health and well being of the people in these regions, as well as the environment.

This research has shown the importance of Economic and Population growth driven water demand uncertainty relative to climate change uncertainty facing water infrastructure investment planning to mid-century. The insights are valuable to policy makers and analysts working on water infrastructure planning. These results do not necessarily imply an insurmountable future for this region. Through climate mitigation, and perhaps most importantly, proper planning and financing of adaptive and protective measures for these anticipated shortages—based on reliable information as to the effectiveness of certain strategies to avert the risks presented above—these future water systems can be augmented to better ensure their resiliency and sustainability. Addressing these options for the future, however, will require substantial research and additional experimentation with integrative tools like the IGSM-WRS. Forthcoming studies will expand upon these experiments to quantify the effectiveness of climate mitigation policies and widespread adaptive measures such as enhanced storage, expansive water transfers, water-use efficiencies, and reduced consumption via water mandates or changes in common practice.
